# L-Carnitine and extendin-4 improve outcomes following moderate brain contusion injury

**DOI:** 10.1038/s41598-018-29430-6

**Published:** 2018-07-25

**Authors:** Hui Chen, Yik Lung Chan, Claire Linnane, Yilin Mao, Ayad G. Anwer, Arjun Sapkota, Tiara F. Annissa, George Herok, Bryce Vissel, Brian G. Oliver, Sonia Saad, Catherine A. Gorrie

**Affiliations:** 1 School of Life Sciences, Faculty of Science, University of Technology Sydney, Broadway, NSW 2007 Australia; 2 Faculty of Basic Medical Sciences, Chengdu University of Traditional Chinese Medicine, Chengdu, Sichuan 610072 China; 3 ARC Centre of Excellence for Nanoscale Biophotonics, Macquarie University, North Ryde, 2109 NSW Australia; 4 School of Applied Sciences, Republic Polytechnic, 738964 Singapore, Singapore; 5 Centre for Neuroscience and Regenerative Medicine, Faculty of Science, University of Technology Sydney, Broadway, NSW 2007 Australia; 6 St Vincent’s Hospital Centre for Applied Medical Research, Darlinghurst, NSW 2010 Australia; 7 Kolling Institute of Medical Research, University of Sydney, St Leonards, NSW 2065 Australia

## Abstract

There is a need for pharmaceutical agents that can reduce neuronal loss and improve functional deficits following traumatic brain injury (TBI). Previous research suggests that oxidative stress and mitochondrial dysfunction play a major role in neuronal damage after TBI. Therefore, this study aimed to investigate two drugs known to have antioxidant effects, L-carnitine and exendin-4, in rats with moderate contusive TBI. L-carnitine (1.5 mM in drinking water) or exendin-4 (15 µg/kg/day, ip) were given immediately after the injury for 2 weeks. Neurological function and brain histology were examined (24 h and 6 weeks post injury). The rats with TBI showed slight sensory, motor and memory functional deficits at 24 h, but recovered by 6 weeks. Both treatments improved sensory and motor functions at 24 h, while only exendin-4 improved memory. Both treatments reduced cortical contusion at 24 h and 6 weeks, however neither affected gliosis and inflammatory cell activation. Oxidative stress was alleviated and mitochondrial reactive oxygen species was reduced by both treatments, however only mitochondrial functional marker protein transporter translocase of outer membrane 20 was increased at 24 h post injury. In conclusion, L-carnitine and exendin-4 treatments immediately after TBI can improve neurological functional outcome and tissue integrity by reducing oxidative stress.

## Introduction

Traumatic brain injury (TBI) can cause a number of cognitive deficits including impaired attention and memory, reduced information processing speed and difficulty in communicating^1,2^. Mild TBI accounts for 70–90% of all hospital-treated TBI^3^. The World Health Organisation has predicted that by the year 2020, TBI will be the third leading cause of death and disability for all ages, impacting not only the individual but the entire community^4^.

During TBI, the mechanical force to the brain initiates a series of biochemical and pathological changes to the affected tissue that can lead to permanent damage and cell death, resulting in neurological deficits that often present as sensorimotor and/or cognitive impairments^5^. Currently, there is no solid evidence suggesting the efficacy of existing treatments for moderate TBI, except for those to alleviate the immediate symptoms, such as anticonvulsants^6^. Therefore, there is a need for therapeutic agents that can prevent excessive neuron loss and speed up tissue repair or regeneration to reduce functional deficits.

Previous research in TBI including ours has found that oxidative stress and mitochondrial dysfunction play a major role in ischemia induced neuronal damage^2,7,8^. The brain functions exclusively under strict aerobic conditions and as such, it is subject to the by-products of aerobic respiration specifically reactive oxygen species (ROS)^9^. ROS at high concentrations can induce degradation of cellular structures leading to impaired cellular function and cell death^10^. The majority of ROS are produced by the electron transport chain, a series of complexes (called Oxidative phosphorylation (OXPHOS) complexes I–V) in the inner mitochondrial membrane that serve to transfer electrons and hydrogen ions across the membrane to produce ATP^11,12^. To ensure the level of ROS stays within a safe range, the cells are equipped with an endogenous antioxidant defence system, such as manganese superoxide dismutase (MnSOD) to scavenge ROS^13^. TBI initiates a number of cascades which induce oxidative stress by either enhancing ROS synthesis or impairing the antioxidant defence mechanisms^14^. As the major source of ROS, the mitochondria are vulnerable and subject to oxidative stress damage and resulting functional disorder during TBI. With the occurrence of a traumatic impact to the brain, an inflammatory response is initiated, resulting in the migration of pro-inflammatory cells to the injury site^2^. Inflammatory cells such as microglia and macrophages contain nicotinamide adenine dinucleotide phosphate oxidase, an enzyme attributed to the reduction of molecular oxygen to form ROS, worsening oxidative stress^15,16^.

Previously, we modelled a unilateral cortical contusion using a weight drop method^2^. The rats with TBI displayed significant cognitive and motor deficit immediately after injury, with increased gliosis and oxidative stress markers, as well as abnormal mitochondrial functional markers^2^. As such, reducing oxidative stress to improve mitochondrial function may be a feasible target for early intervention to ameliorate tissue damage and functional loss following mild TBI. L-Carnitine and Exendin-4 have shown to have antioxidative effects and neuroprotective effects in conditions of impaired neurological function^17–22^. The major obstacle of treating brain injury by pharmacological means is the blood brain barrier, which both L-Carnitine and Exendin-4 can easily cross. Therefore, the aim of this study was to investigate whether early treatment using these two medications can mitigate tissue damage and functional decline after moderate TBI in a rat model.

## Animal and Materials

### Modelling mild brain cortical contusion

The animal experiments were approved by the Animal Care and Ethics Committee (ACEC# 2014-478) at the University of Technology Sydney, following the guidelines by the National Health and Medical Research Council of Australia.

Female Sprague-Dawley rats (~250 g, n = 48, Animal Resource Centre, Western Australia, Australia) were given one week to acclimatise to the facility with free access to water and standard rodent chow (Gordon’s Specialty Stockfeeds, NSW, Australia) in a room on a 12:12 hour light-dark cycle.

The rats were randomly divided into four groups; sham (n = 12), TBI-only (n = 12), TBI with L-Carnitine treatment (TBI + LC, n = 12), and TBI with Exendin-4 treatment (TBI + Exd-4, n = 12). Animals were anesthetised with 4% isoflurane (1% oxygen) and maintained at 2% isoflurane (1% oxygen). A craniectomy was made on the right side of the skull 2.5 mm posterior and 3.0 mm lateral to bregma using a dental drill. A 10 g rod with a 2.5 mm diameter impactor head, was dropped from 5 cm onto the surface of the brain using a New York University Impactor as previous published^2^. This produces a moderate brain injury, with a small focal lesion. The contusion was confirmed visually in all TBI rats. Bone wax was placed over the skull hole and the skin sutured closed. Sham rats did not undergo the weight drop impact, but all other procedures were identical. Immediately after the surgery, the TBI rat received LC (1.5 mM in drinking water) or Exendin-4 (15 µg/kg, once daily, ip, Auspep, VIC, Australia) for 14 days. The doses of LC and Exendin-4 were adopted from our previous studies in the other conditions^23,24^. Analgesics (Buprenorphine hydrochloride-Temgesic 0.03 mg/kg s.c.), antibiotics (Cefazolin sodium 33 mg/kg s.c.) and Hartmans replacement solution (Compound sodium lactate 15 ml/kg s.c.) were given prior to the surgery and every 12 hours for 3 days post-surgery. Half of each group were harvested at 24 hrs post-surgery, while the other half were harvested at 6 weeks post-surgery as pervious published^2^. Rats were culled after anaesthetic overdose with Lethobarb (pentobarbitone, 1 ml/100 g, i.p). The brain tissues were collected for histology, western blotting and rt-PCR analysis as previous described, resulting in sample sizes of n = 3–6 for each outcome measure^2^.

### Behavioural tests

Animals were assessed for cognition (novel object recognition (NOR) test)^25^, forelimb motor function (Error Ladder Walking test)^26^, anxiety (elevated plus maze test) and sensory function (sticky tape removal test^27,28^) at 24 h, 1 week and 6 weeks after the surgery. All the video analysis was performed by two researchers blind to the groups.

#### Sticky Tape test

A circular sticker (20 mm in diameter) was attached to the lower region of the phalanges and hairless part of the forepaw (the metacarpal pads, thenar, and hypothenar). Shaking of the limb, looking at, or touching the adhesive with the other paw or nose/whiskers was considered acknowledgement of the sticker. The time for the rat to acknowledge the sticker was recorded. The result is expressed as the ratio of the time of left over the right paw to acknowledge the sticker^29^. A ratio less than one signified the affected left paw was more sensitive than the normal right side and a ratio greater than one indicated the left paw was less sensitive to the adhesive than the right side.

#### Error Ladder Walking test

The rat walked on a horizontal ladder with uneven rungs for 2 minutes. The stepping error was expressed as a percentage calculated from the number of left fore paw stepping errors compared to the number of total steps within the 2 minutes.

#### Elevated plus maze test

The rat was placed at the intersection of the plus maze and video-recorded for two minutes. The percentage of the test time spent in the open arms served as an indicator of the calmness of the rat; whereas an anxious rat would spend a more time in the closed arms^30^.

#### NOR test

The test was set up as we have previously published^31^. All rats were habituated to the testing apparatus (dark-wall chamber with floor space floor 40 × 29 cm^2^) for 1 week prior to the surgery. During the NOR test, each rat underwent a familiarisation phase (5 mins in the chamber exposed to two identical objects (blue square blocks)). They were then returned to the home cage for 1 hour before being returned to the chamber for the test phase (5 mins exposed to one old (blue square block) and one novel (orange triangular block) object. Both sessions were digitally captured for later analysis and the time spent investigating each block was recorded. All data for the NOR tests is presented as the percentage of time spent on the novel blocks in the test stage.

### Histology and immunohistochemistry

Frozen brain tissues were sectioned (15 µm) in the coronal plane and stained with hematoxylin and eosin (H&E) prior to immunohistochemistry on adjacent sections. Lesion analysis was carried out by assessing the extent of haemorrhage, tissue disruption and loss from the cortex, hippocampus and thalamus of the right hemispheres (0, No obvious signs of lesion; 1, <10% area showing haemorrhage, tissue disruption and loss; 2, 10–40% area showing haemorrhage, tissue disruption and loss; 3, >40% area showing haemorrhage, tissue disruption and loss). The overall score is a cummulative score out of 9 for each brain.

Immunohistochemistry was carried out using the following primary antibodies: rabbit anti-GFAP (1:1000, Dako, Denmark) to label astrocytes and mouse anti-CD68 (Ectodysplasin A (ED1), 1:2000, AbD Serotec, Germany) to label macrophages. Prior to the primary antibody incubation, slides were incubated in 5% normal goat serum in phosphate buffered saline with Triton X-100 at pH 7.4 (PBST) for 30 minutes. Primary antibodies were diluted with 5% normal goat serum in phosphate buffer (PBG) and incubated overnight at 4 °C. Slides were washed in PBST and incubated in goat anti-rabbit Alexa Fluor 488 (1/200, Invitrogen, USA) or goat anti-mouse Alexa Fluor 568 (1/200, Invitrogen, USA) in 5% PBG for 2 hours at room temperature. Slides were washed with PBST and counterstained by Hoechst (1:5000, Invitrogen, USA) for 10 minutes. All slides were cover slipped in fluorescent mounting medium (Dako, Denmark). Imaging of immunohistochemistry was carried out using an Olympus BX-51 microscope with an Olympus U-RFL-T fluorescence burner.

Image analysis was carried out on samples taken from the cortex, hippocampus and thalamus of the left and right hemispheres, using ImageJ software (National Institutes of Health, version 2X). Analysis of astrocytes was undertaken by measuring the staining intensity (mean grey scale value) of GFAP in one low power field of view for each brain region near the center of the lesion after normalising against the background for each section using the internal features of the software. ED1 positive cells were counted in each brain region at the same level as for GFAP in six high power fields (ROI = 2786 µm^2^) taken from the edge of the lesion for TBI, the hippocampus and the thalamus directly beneath the lesion or at the equivalent location for shams.

Brain apoptosis and DNA damage were assessed in the formalin fixed frozen sections using active caspase-3 and TUNEL staining (n = 3–5). Cortex, hippocampus and thalamus were accessed. For all the staining, the sections were hydrated gradually from xylene followed by changes of graded ethanol to distilled water.

For active caspase-3 and TUNEL staining, the sections underwent heat-induced epitope retrieval by microwaving (LG, Australia) for 13 mins in citric antigen retrieval buffer (pH 6.0) followed by cooling in a water bath for 15 mins.

For active caspase-3 staining, the tissues were incubated with active caspase-3 antibody (1:800, Cell Signalling Technology, USA) dilution using EnVision^TM^ FLEX antibody diluent (Dako, Denmark) at room temperature for one hour. Negative controls were incubated with only the antibody diluent. The sections were then incubated with labelled polymer-HRP Anti-rabbit (Dako, Denmark) for one hour and then visualized using DAB (Dako, Denmark). Sections were subsequently counterstained with hematoxylin and coverslipped.

ApopTag® Peroxidase kit (S7100, Merck Millipore, VIC, Australia) was used for TUNEL staining. Sections were incubated with 50ul of equilibration buffer for 30 s, and coverslipped after the hydration step. Terminal deoxynucleotidyl transferase (TdT, 25ul, Tdt: reaction buffer = 1:4) was added to each section, coverslipped and incubated for one hour at 37 °C. Negative controls were incubated with water instead of Tdt. The coverslip was then removed and sections inserted in stop reaction buffer for 10 mins before incubation in anti-digoxigenin-peroxidase for 40 mins at room temperature, followed by DAB (Dako, Denmark) for color development, counterstaining in hematoxylin and coverslipping.

For both active caspase-3 and TUNEL, the number of positive stained neurons was manually counted in the cerebral cortex, hippocampus and thalamus. Imaging was conducted using NanoZoomer Slide Scanner (Hamamatsu Photonics, Japan) with a 20X objective. Positive neuron density was presented as the percentage of positively stained neurons (brown) among total number of cells counterstained by hematoxylin (blue).

### Mitochondrial density and ROS

Mitochrondria were visualised 24 hours post-injury using MitoTracker Green (Thermo Fisher Scientific, Australia) at 200 nM final concentration and images were acquired at 488 nm excitation wavelength and detected in the 510–550 nm emission range. For total reactive oxygen species (ROS) detection, CellROX Deep Red (Thermo Fisher Scientific, Australia) was used at 5 µM final concentration and images were acquired at 633 nm excitation wavelength and detected in the 640–680 nm emission range. Confocal laser scanning microscopy images of frozen brain sections were acquired using Leica SP2 confocal laser scanning microscope (Leica, Wetzlar, Germany). Data was generated from 4–5 animals/group. Three images were collected from each cortex and averaged before the analysis was quantified using ImageJ. All imaging parameters including laser intensities, Photomultiplier tubes voltage and pinholes were kept constant during imaging.

### Real-time PCR

Total mRNA was extracted from brain tissues using TriZol reagent (Life Technologies, CA, USA). M-MLV Reverse Transcriptase, RNase H, Point Mutant Kit (Promega, WI, USA) was used to generate first-strand cDNA using purified total RNA^32^. Genes of interest were measured using manufacturer pre-optimized and validated iNOS Taqman® primers and probes (Thermo Fisher Scientific, CA, USA, probe sequence: GGCCTTGTGTCAGCCCTCAGAGTAC). The probes of the iNOS were labelled with FAM^®^ dye and those for housekeeping 18s rRNA were labelled with VIC^®^ dye. Gene expression was standardized to 18s RNA. The average expression of the control group was assigned as the calibrator against which all other samples are expressed as fold difference.

### Western Blotting

Brain tissue was homogenised in cell lysis buffer for whole protein and mitochondrial protein extraction as previously described^23,33^. The protein levels of antioxidant manganese superoxide dismutase (MnSOD) and protein transporter translocase of outer membrane (TOM)20 proteins were measured in brain lysis; while dynamic-related protein (DRP)−1 and optic atrophy (OPA)−1 were measured in the mitochondria. Protein samples of 40 µg were separated on NuPage® Novex® 4–12% Bis-Tris gels (Thermofisher, CA, USA) and then transferred to PVDF membranes (Rockford, IL, USA). Membranes were then blocked with non-fat milk powder and incubated with primary antibodies against (MnSOD (1:1000) and TOM20 (1:2000), Santa Cruz Biotechnology, Texas, USA); Mitoprofile Total® OXPHOS complex Rodent WB cocktail antibody (1:2500, Abcam); DRP-1 (1:2000, Novus Biologicals, CO, USA), OPA1–1 (1:2000, Novus Biologicals, CO, USA) for overnight and then goat anti-rabbit or rabbit anti-mouse IgG horseradish peroxidase-conjugated secondary antibodies (Santa Cruz Biotechnology, 1:5000 for MnSOD, TOM20) as we have previously published^34,35^. Protein expression was detected by SuperSignal West Pico Chemiluminescent substrate (Thermo Fisher, MA, USA) by exposure of the membrane in FujiFlim (Fujifilm, Tokyo, Japan). Protein band density was determined with Image J software (NIH, MD, USA).

### Statistical method

The results are expressed as mean ± SE. The difference between the groups was analysed by one-way ANOVA followed by Turkey post hoc test (Statistica 9, Statsoft, USA). The results of brain lesion grade were analysed by Kruskal-Wallis test with Dunn’s multiple comparisons test. P < 0.05 was considered significant.

## Results

### Behavioural changes

#### Sticky-Tape Test

**Figure 1:**
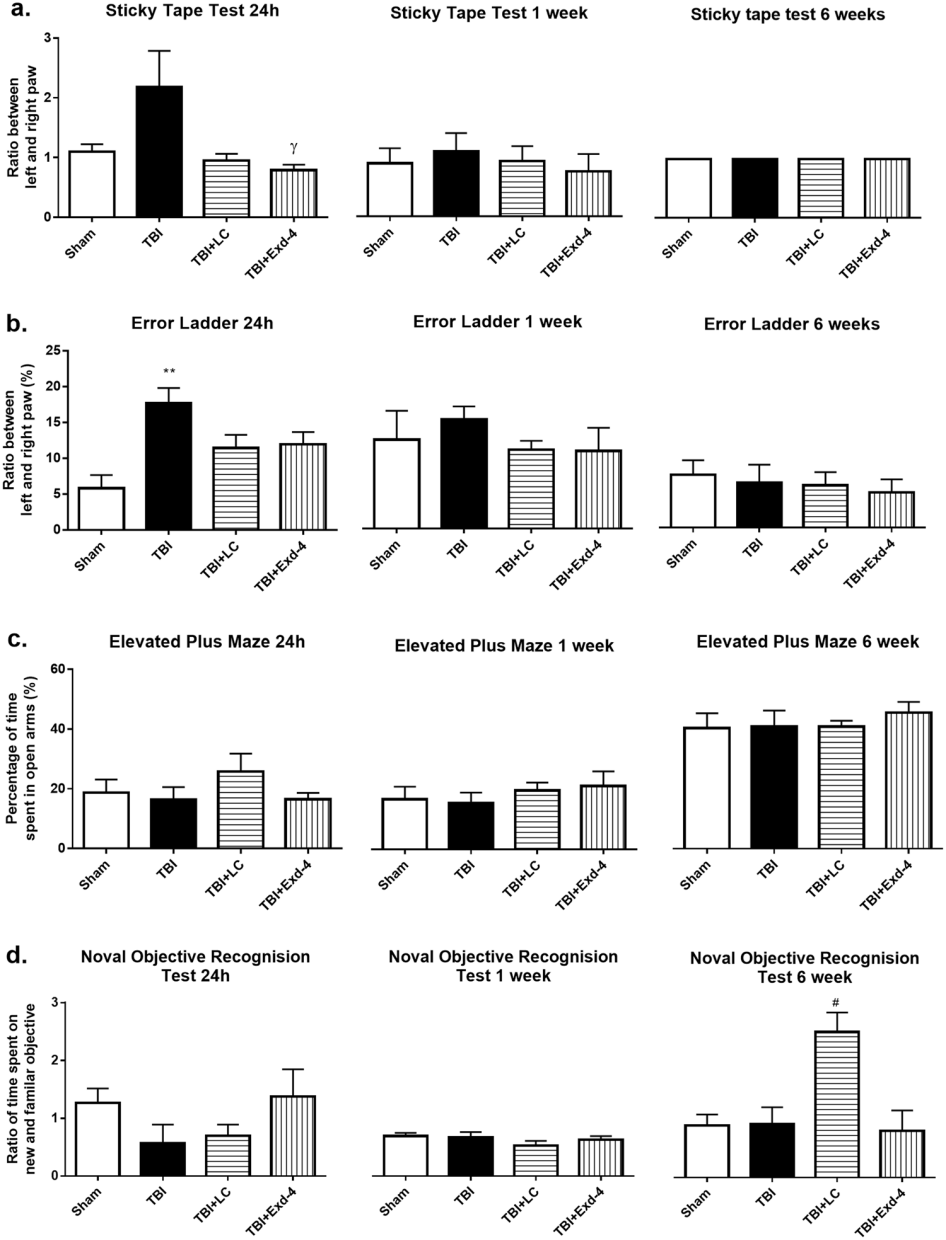
Behaviour outcome in Sticky Tape Test (**a**) Error Ladder Test (**b**) Elevated Plus Maze Test (**c**) Noval Objective Recognision Test (**d**) in rats at 24 hours, 1 week and 6 weeks post-surgery. Results are expressed as mean ± SEM, n = 12 at 24 hours and n = 6 at 1 week and 6 weeks. Data was analysed by One-way ANOVA with Bonferroni *post hoc* tests. **P < 0.01 *vs* Sham; ^γ^P < 0.05 *vs* TBI; ^#^P < 0.05 *vs* all 3 groups. TBI: Traumatic brain injury; TBI-LC: Traumatic brain injury with L-Carnitine treatment; TBI-E: Traumatic brain injury with Exendin-4 treatment.

At 24-hour post-surgery, as expected the ratio in the TBI-only rats was doubled compared with the Sham group albeit without statistical significance (Fig. 1a), suggesting reduced sensation in the left paw; whereas the ratio was normalised to the Sham level in L-Carnitine and exendin-4 treated rats (P < 0.05 TBI-E vs TBI, Fig. 1a). From 1 week onward, there was no difference among the four groups.

#### Error Ladder Test

At 24 hours, the rats in the TBI group made more than 3 times the percentage of left forepaw stepping errors than the Sham rats (P < 0.01, Fig. 1b). The percentage of stepping errors was much less in two treatment groups compared to the TBI group, although not statistically significant (Fig. 1b). Both treatments had similar effects to improve left forepaw locomotion. The motor deficit seems to be recovered at 1 week.

#### Elevated Plus Maze Test

There was no difference on the percentage of time spent on the open arm at any time point (Fig. 1c). However, the time was doubled in all four groups at 6 weeks, which may be due to repeated exposure which made the rats less anxious on the open arm.

#### Novel Object Recognition test

At 24 hours, the Sham group spent slightly more time on the new objection, while it was halved in the TBI rats (Fig. 1d). Rats in the TBI-LC group showed similar performance as the TBI rats, while rats in the TBI-E group behave similarly as the Sham rats although without statistical significance (Fig. 1d). The performance was similar among all groups at 1 week (Fig. 1d). TBI-LC rats showed significantly increased interest in the new objective at 6 weeks (P < 0.05 *vs* all the other 3 groups); whereas the other 3 groups were similar (Fig. 1d).

### Brain lesion morphology

**Figure 2:**
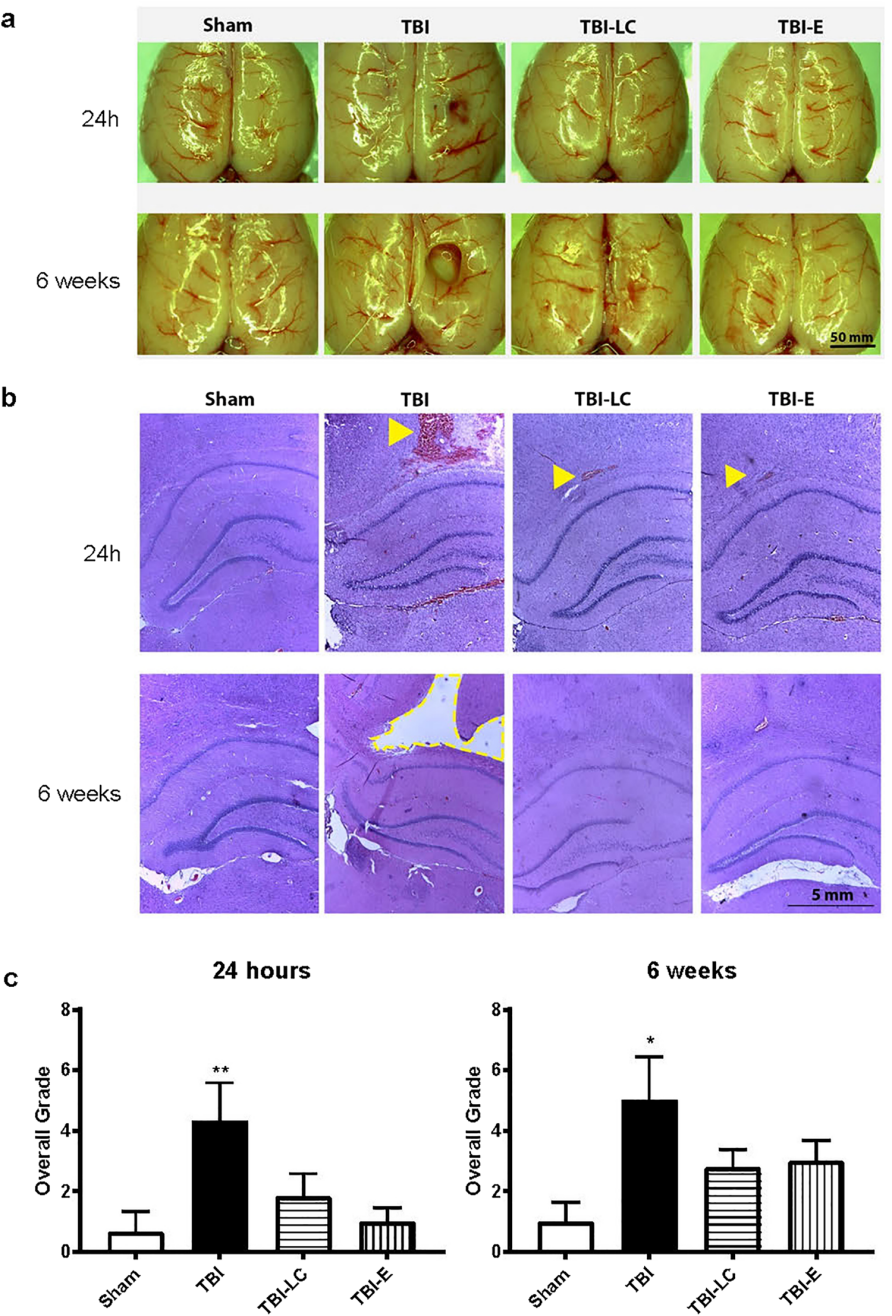
Dorsal images of brain (**a**), representative haematoxylin and eosin-stained coronal sections of the right brain at lesion level (**b**), and semi-quantification of injury in the coronal brain sections by overall grade (**c**) at 24 hours and 6 weeks post-surgery. In (**b**), signs of injury included tissue disruption, haemorrhage (arrowheads) and tissue loss (dotted line) presented as a cavity (TBI at 6 week). Data was analysed by One-way ANOVA with Bonferroni *post hoc* tests, n = 6, *P < 0.05, **P < 0.01 vs Sham. TBI: Traumatic brain injury; TBI-LC: Traumatic brain injury with L-Carnitine treatment; TBI-E: Traumatic brain injury with Exendin-4 treatment.

There was no cortex contusion in the Sham rats at 24 hours and 6 weeks post-surgery (Fig. 2a). The contusive injury was located at the right dorsal surface of the brain (Fig. 2a). In the TBI group, focal haemorrhage was observed at 24 hours following surgery and a cavity formed at the lesion site by 6 weeks (Fig. 2a). There were no distinct signs of injury at the dorsal surface of the injured brains treated with L-Carnitine or Exendin-4 at both time points (Fig. 2a). The coronal brain sections stained by H&E demonstrated the contusive injury mainly located in the cortex layer, slightly extending to the hippocampus layer (Fig. 2b). The haemorrhage and tissue disruption was visible in all injured brains at 24 hours post-injury and the tissue loss represented as a cavity in the TBI group at 6 weeks post-injury. The injury was semi-quantified and shown in overall grade of injury according to the extent of haemorrhage, tissue disruption and loss from the cortex, hippocampus and thalamus layers at 24 hours (Fig. 2b) and 6 weeks (Fig. 2c) following injury. The overall grade of injury for the TBI rats was significantly higher than that for the Sham rats at 24 hours injury (P < 0.01). The L-Carnitine and Exendin-4 treatments reduced the overall grade of injury to less than half that of TBI group at 24 hours. By 6 weeks post-injury, the overall grade of injury in TBI group was still significantly higher than that in the Sham group (P < 0.05), which was lower in L-Carnitine and Exendin-4 treated rats (Fig. 2c).

**Figure 3:**
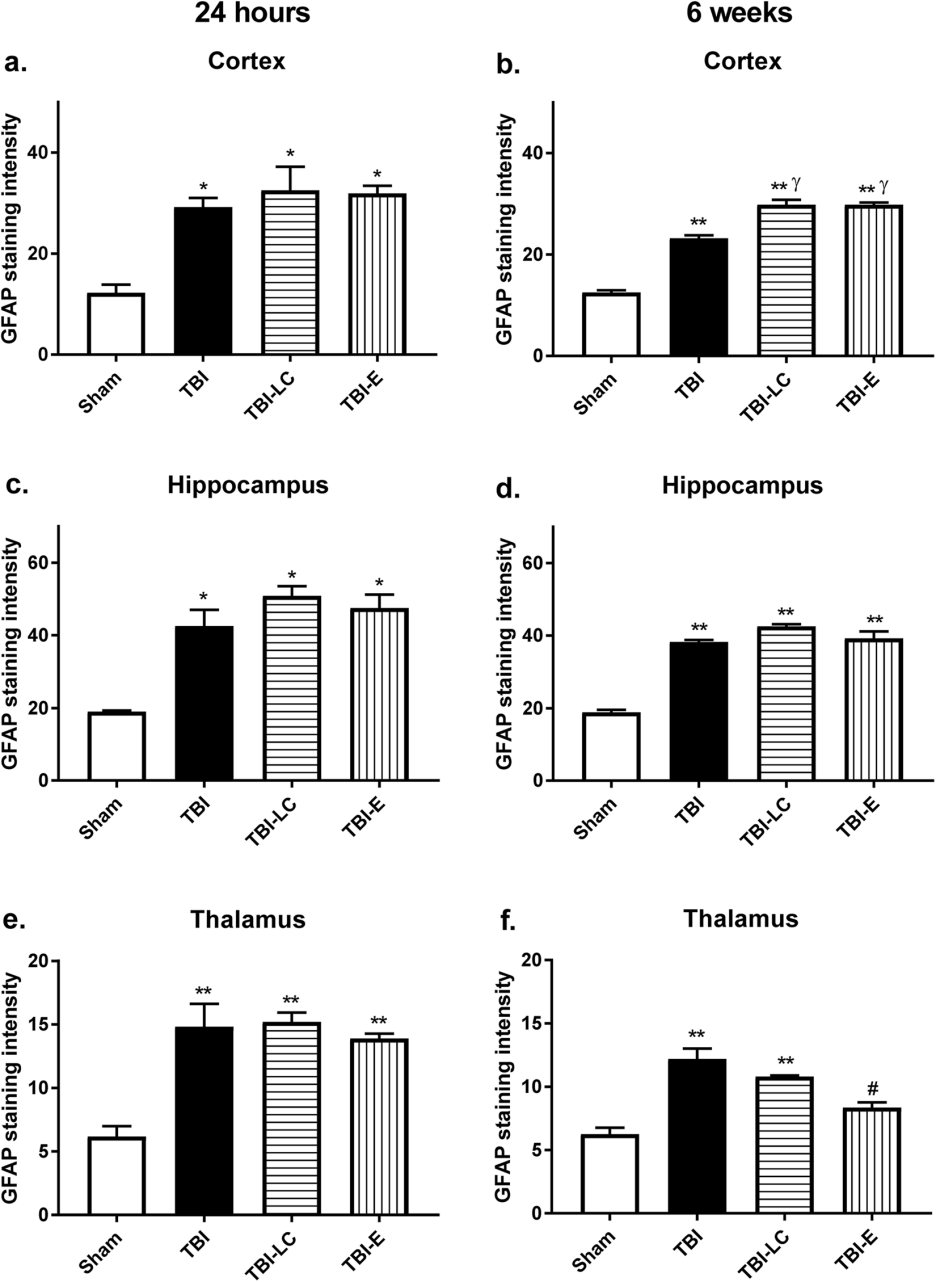
Glial Fibrillary Acidic Protein (GFAP) positive staining intensity as the mean greyscale value in the right (**a**) cortex, (**b**) hippocampus and (**c**) thalamus at 24 hours and 6 weeks post injury. Data was analysed by One-way ANOVA with Bonferroni *post hoc* tests, n = 3–6, *P < 0.05, **P < 0.01 *vs* Sham; ^γ^P < 0.05 *vs* TBI group; ^#^P < 0.05 *vs* all the other 3 groups.

At 24 hours, GFAP positive cells were significantly increased in all rats with brain injury regardless of the treatment in all 3 regions measured (P < 0.05 *vs* Sham in the cortex and hippocampus; P < 0.01 *vs* Sham in the thalamus, Fig. 3a,c,e). At 6 weeks, there were significantly more GFAP positive cells in the TBI rats (P < 0.01 *vs* Sham, Fig. 3b), and this was increased in the L-Carnitine and Exendin-4 treated rats (both P < 0.05 *vs* Sham, Fig. 3b). GFAP cell changes in the hippocampus were similar as those at 24 hours (all 3 groups P < 0.01 *vs* Sham, Fig. 3d). In the thalamus, GFAP cell was doubled in TBI and TBI-LC rats (both P < 0.01 *vs* Sham, Fig. 3f), which was however reduced in Exendin-4 treated rats (P < 0.05 *vs* Sham, TBI and TBI-LC, Fig. 3f).

**Figure 4:**
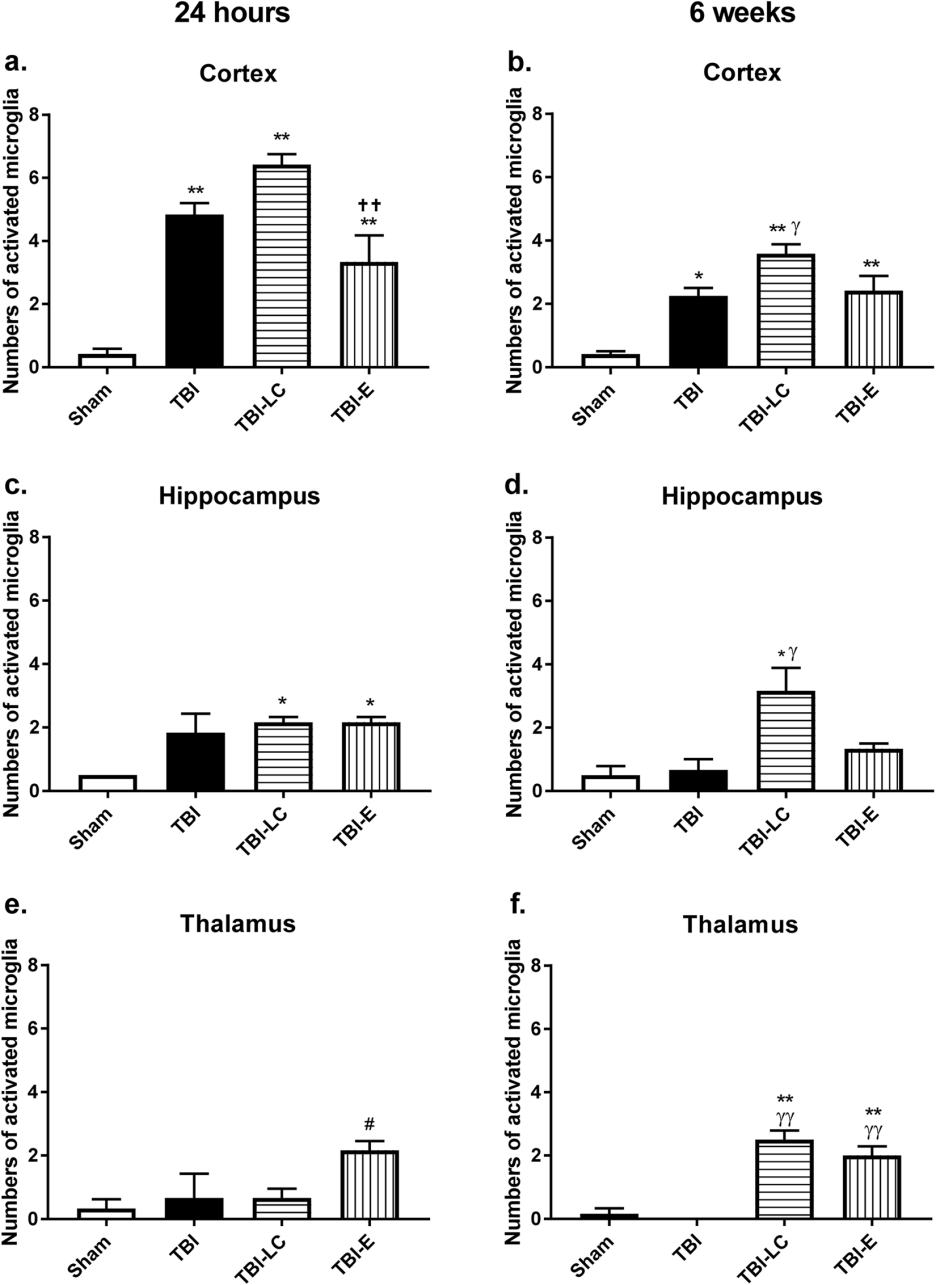
Activated macrophages/microglia cell number reflected by Ectodysplasin A (ED1)/Iba1 double positive staining in the right (**a**) cortex, (**b**) hippocampus and (**c**) thalamus at 24 hours and 6 weeks post injury. Data was analysed by One-way ANOVA with Bonferroni *post hoc* tests, n = 3–6, *P < 0.05, **P < 0.01 *vs* Sham; ^γ^P < 0.05, ^γγ^P < 0.01 *vs* TBI group ^††^P < 0.01 *vs* TBI-LC; ^#^P < 0.05 *vs* all the other 3 groups.

At 24 hours, there were markedly increased activated microglia cells reflected by ED1 positive staining in both cortex and hippocampus in rats with brain injury regardless of the treatment (Fig. 4a,c). In the thalamus, only TBI-E rats had significantly increased activated microglia cells (P < 0.05 *vs* the other 3 groups, Fig. 4e). At 6 weeks, there were higher levels of activated microglia cells in TBI and TBI-E groups (P < 0.05, P < 0.01 *vs* Sham respectively), and these were increased in the TBI-LC group (P < 0.05 *vs* TBI, Fig. 4b). In the hippocampus, only TBI-LC rats had more activated microglia cells (P < 0.05 *vs* Sham and TBI, Fig. 4d), whereas in the thalamus, both TBI-LC and TBI-E rats had increased number of activated microglia cells (P < 0.01 *vs* Sham and TBI, Fig. 4f).

### Inflammatory and oxidative stress markers

**Figure 5:**
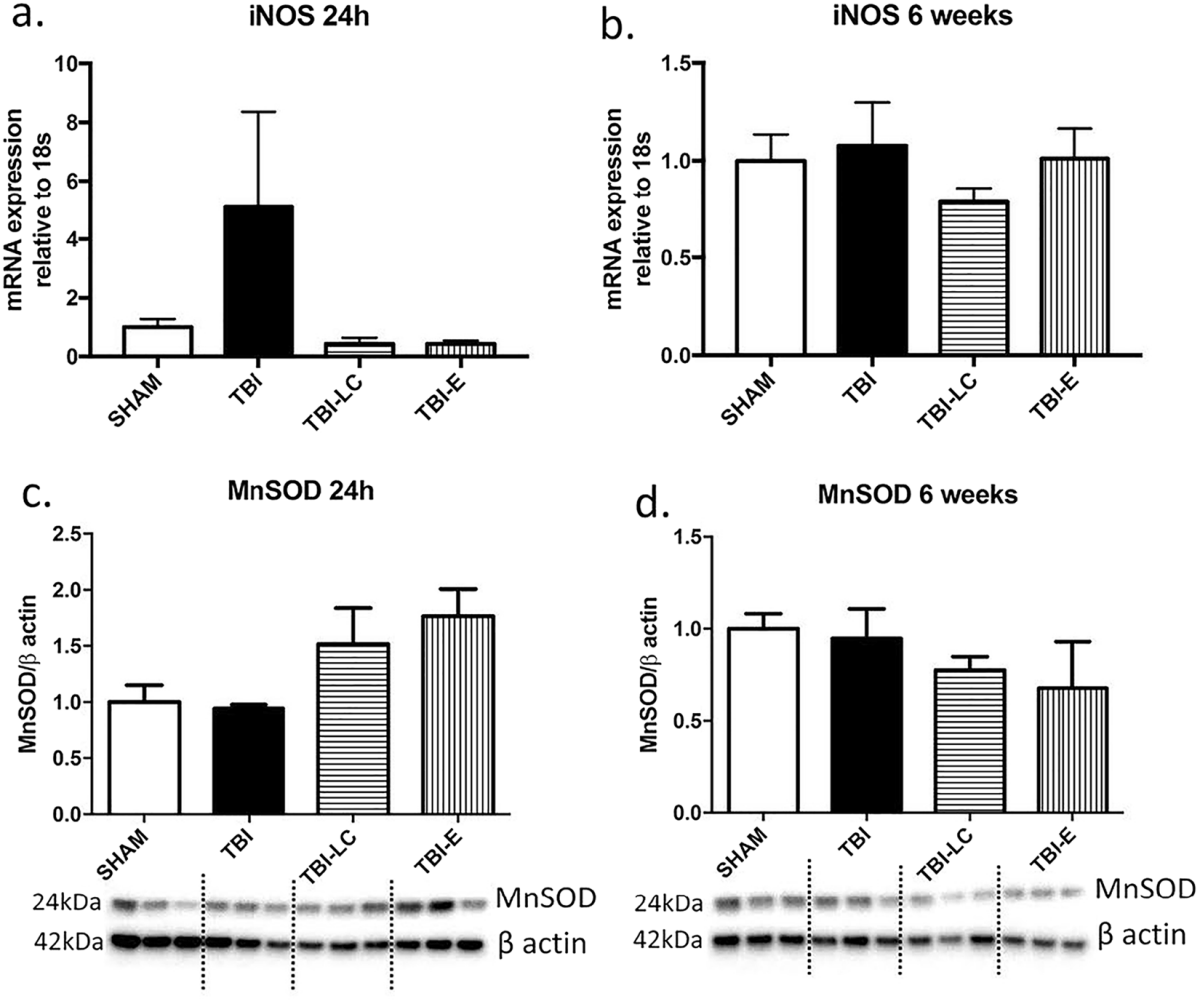
mRNA expression of iNOS (**a**,**b**) and protein levels of mitochondrial oxidative stress marker MnSOD (**c**,**d**) at 24 h and 6 weeks post-injury. Data was analysed by One-way ANOVA with Bonferroni *post hoc* tests, n = 3–6, Results are expressed as mean ± SE. Whole gel images in Supplementary Fig. 1.

At 24 h post injury, brain iNOS expression was markedly increased in the TBI brains albeit without statistical significance (Fig. 5a). The levels of iNOS in the two treatment groups were similar to Sham group (Fig. 5a). MnSOD protein levels were increased by more than 50% in both treatment groups although without statistical significance (Fig. 5c). At 6 weeks, there was no difference in iNOS nor MnSOD levels among the groups (Fig. 5b,d).

**Figure 6:**
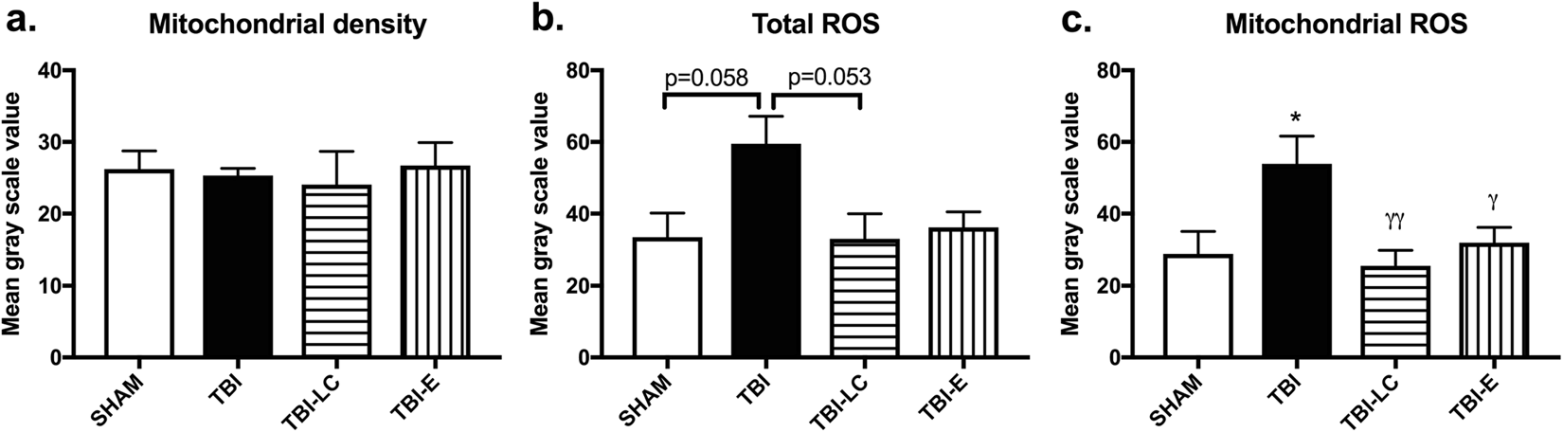
Mitochondrial density (**a**), total ROS (**b**), and mitochondrial ROS (**c**) levels in the brain cortex at 24 h post-injury. Results are expressed as mean ± SE. Data was analysed by One-way ANOVA with Bonferroni *post hoc* tests, n n = 3–6, *P < 0.05 *vs* Sham; ^γ^P < 0.05, ^γγ^P < 0.01 *vs* TBI.

Mitochondrial density was unaffected by either the traumatic injury itself, or by the treatment with anti-oxidant drugs, however mitochondrial ROS increased after injury and were reduced after treatment with both L Carnitine and with Exendin-4. (Fig. 6).

### Mitochondrial functional and integrity markers

**Figure 7:**
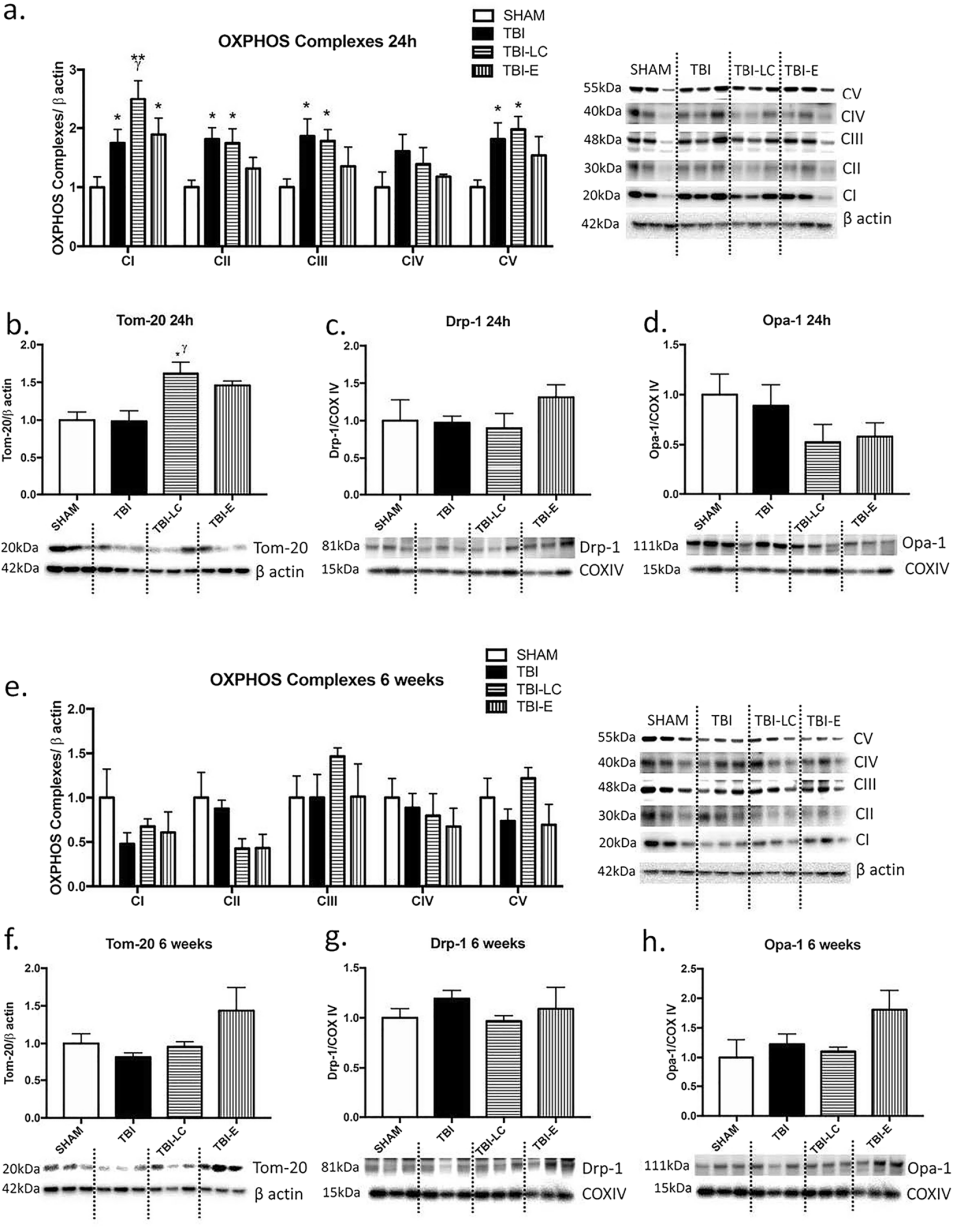
Protein levels of mitochondrial functional marker OXPHOS complexes I-V (**a**,**e**) and Tom-20 (**b**,**f**), and mitophagy markers DRP-1 (**c**,**g**), and Opa-1 (**d**,**h**) at 24 h and 6 weeks post-injury. Results are expressed as mean ± SE. Data was analysed by One-way ANOVA with Bonferroni *post hoc* tests, n = 3–6, *P < 0.05, **P < 0.01 *vs* Sham; ^γ^P < 0.05 *vs* TBI. Whole gel images in Supplementary Fig. 2–9.

At 24 hours, there was a trend of increase in OXPHOS complex I-V in TBI rats, while Exendin-4 treatment seems to normalise it to SHAM level (Fig. 7a). Tom-20 was increased in two treatment groups however only significant in L-Carnitine treated rats (P < 0.05 TBI-LC vs Sham and TBI, Fig. 7b). Mitophagy marker Drp-1 was only increased by 35% in Exendin-4 treated rats (P = 0.62 vs TBI, Fig. 7c), while Opal-1 proteins were nearly halved in injured rats with L-Carnitine and Exendin-4 treatments (Fig. 7d).

At 6 weeks, although OXPHOS complex I was halved in TBI rats and complex II was halved in both treatment groups, they did not reach statistical significance (Fig. 6e). There is a trend of L-Carnitine to normalise complex V level in rats with TBI (Fig. 6e). Tom-20 and Opa-1 levels were only increased in Exendin-4 treated rats although without statistical significance (Tom-20 P = 0.13, Opa-1 P = 0.38 vs TBI, Fig. 7f,h).

### DNA damage and apoptosis

**Figure 8:**
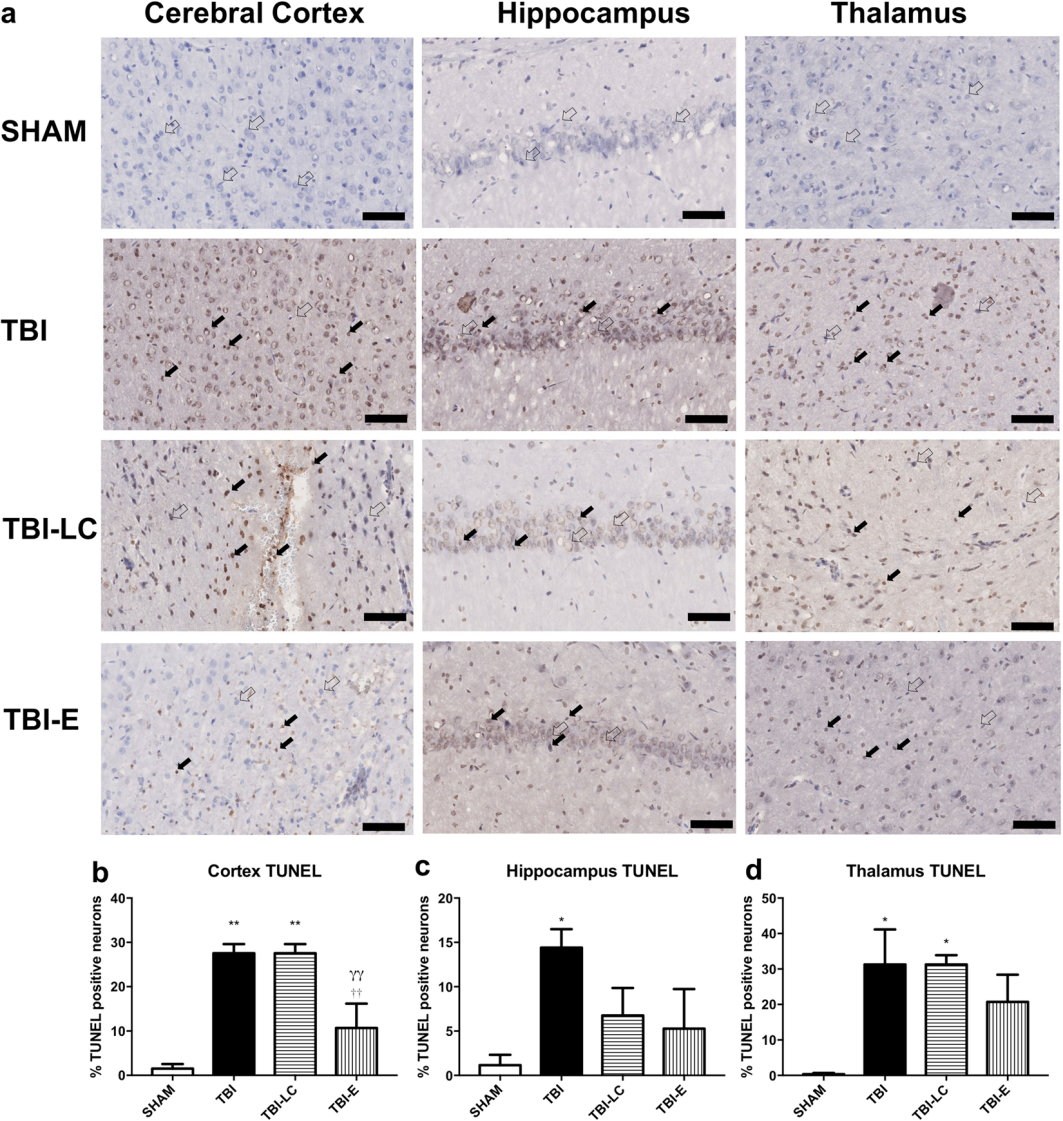
TUNEL staining in cerebral cortex, hippocampus and thalamus (n = 3–5) (**a–d**). TUNEL positive (closed arrow) and TUNEL negative (open arrow). Scale bar = 100 μm. Results are expressed as mean % TUNEL positive neurons ± SE. Data was analysed by One-way ANOVA with Bonferroni *post hoc* tests, n = 3–6, *P < 0.05, **P < 0.01 *vs* Sham; ^γγ^P < 0.01 *vs* TBI; ^††^P < 0.01 *vs* TBI-LC.

**Figure 9:**
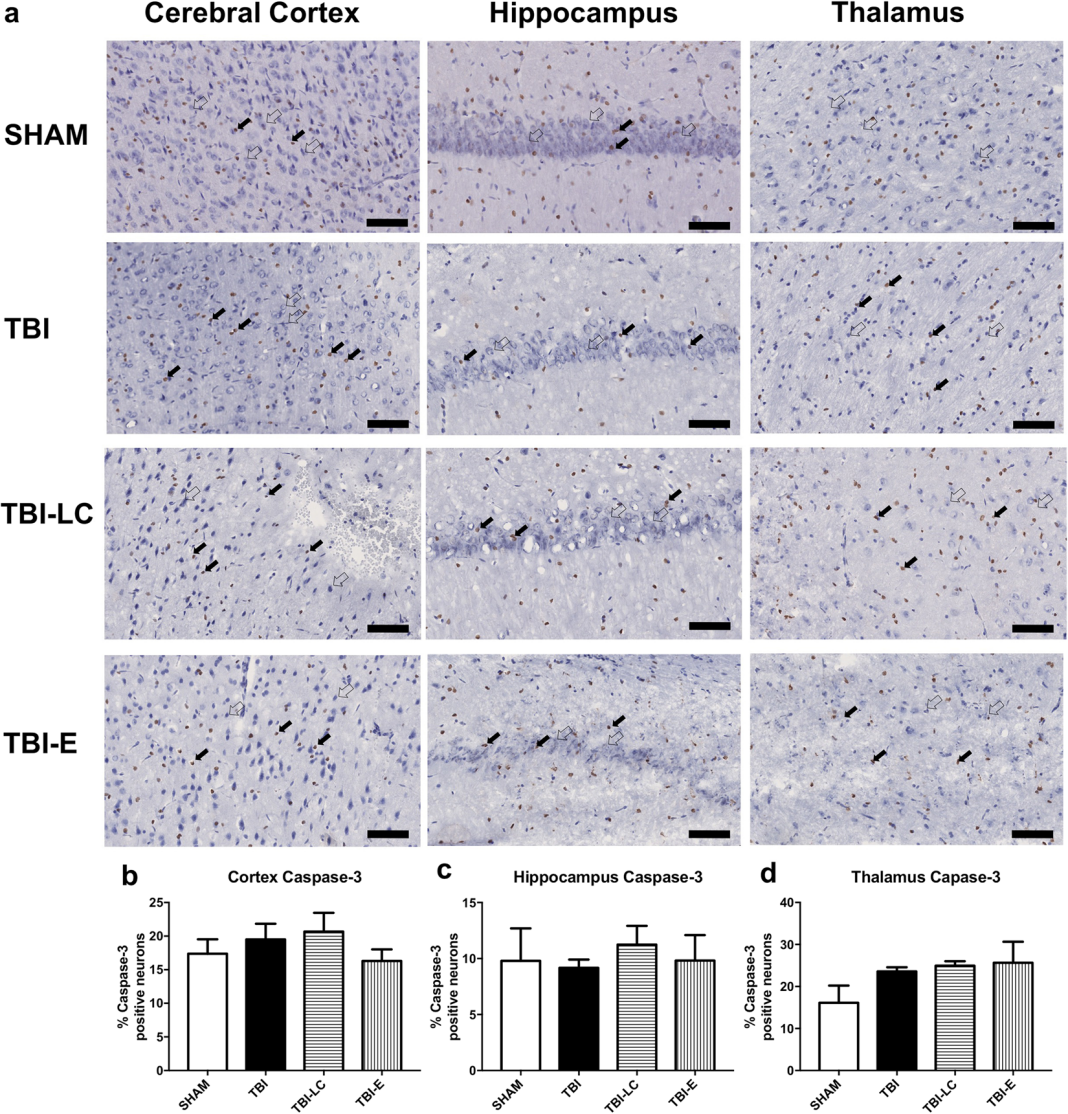
Active caspase-3 staining in cerebral cortex, hippocampus and thalamus (**a–d**). Caspase-3 positive (closed arrow) and TUNEL negative (open arrow). Scale bar = 100 μm. Results are expressed as mean % TUNEL positive neurons ± SE. Data was analysed by One-way ANOVA with Bonferroni *post hoc* tests, (n = 3–5).

TUNEL staining showed the level of DNA damage. TBI increased DNA damage in cortex, hippocampus and thalamus (P < 0.05 Fig. 8). L Carnitine did not affect DNA damage in any of the area. Exendin-4 reduces DNA damage only in cortex (P < 0.01 Fig. 8b). Apoptosis, reflected by active caspase-3 staining remains unaffected by either traumatic injury itself, or by the treatment (Fig. 9).

## Discussion

The available medications to ameliorate tissue damage and any neuropsychiatric consequence following mild TBI only aim to temporarily alleviate the symptoms^36^. In this study, we showed that two existing medications, L-carnitine and Exendin-4, administered immediately after injury significantly ameliorated tissue damage and improved the slight sensorimotor functional deficits seen in rats with moderate TBI. The mechanism seems to lie in alleviated oxidative stress. However, neither of them showed significant impact on mitochondrial functional and integrity markers measured in this study.

Even for moderate TBI, such as the cortical contusion used in this study, tissue damage and cell loss are inevitable. Astrocytes and glia/macrophages are activated in this situation to clear damaged cells, promote tissue repair and scar formation. As neurons do not regenerate spontaneously, the lesion underwent liquefactive necrosis and was transformed into a liquid viscous mass with clear fluid in the cortex, as shown in rats at 6 weeks post injury in this study.

Neural loss is commonly linked to sensorimotor deficits and cognitive impairment in both humans and animal models, especially immediately after the injury occurs^37^. A contusion on the cortex was sufficient to lead to significant impairment of walking coordination in rats at 24 h, with reduced sensing ability in the contralateral forepaw. However, such sensorimotor deficits recovered rapidly, which may be due to the compensation of the motor and sensory cortex on the contralateral side. However, even without significant structural loss in the hippocampus, short term memory function is slightly reduced in the TBI rats at 24 h post injury, reflected by their performance in the novel objective recognition test. Although the data did not show statistical significance, the physiological implication can’t be neglected. Indeed, memory loss and reduced information processing speed due to TBI are well documented in the literature^38,39^. Although tissue loss was significant at 6 weeks, cognitive impairment was fully recovered at this time point. A previous study has found that TBI-induced memory impairment can be persistent, associated with unemployment and difficulties in performing financial management tasks, which significantly affecting employability and life quality^36^. Such observation in humans can be due to continuous neural degeneration, which exacerbate cognitive decline in the long term, whereas the timeline of the current study is too short to observe such changes^40^. Anxiety is a common psychological disorder after a TBI especially in those with multiple TBI^41^. However, human studies only focused on patients with clinically diagnosed TBI, whereas the majority of patients with mild TBI do not necessarily obtain medical attention due to mild nature of the symptoms. Thus, we did not observe any change in anxiety like behaviour in rats with TBI, perhaps because the injury was too mild to cause a measurable change.

Astrocytes are most abundant and important supporting cells in the CNS, critical to maintain the normal functions of the neurons^42^. Within hours post-injury, astrocytes become reactive to form astrocytic scar (called astrogliosis) in response to the mechanical destruction of neural cells for tissue repair^42^. The astrocytic scar acts as a neuroprotective boundary restricting inflammation and limiting lesion expansion^43,44^. Astrocytes are also important for angiogenesis after the injury to restore blood supply to the injured area^45^. However, this scar also creates a physical and chemical barrier that prevents neural and axonal regeneration^42^.

Microglia are the resident macrophages in the CNS whose primarily function is to protect the CNS by engulfing myelin debris and invading micro-organism, known as phagocytosis. At the early stage, the activated microglia are believed to cause neuronal and glial toxicity, as well as neutrophil infiltration by releasing pro-inflammatory cytokines^46^. Indeed, increased activitatin of microglia was displayed in the cortex and hippocampus at 24 h in TBI rats. At the later stage, microglia are suggested to help with neuronal survival by secreting growth factors, such as glial cell line-derived neurotrophic factor and brain-derived neurotrophic factor and anti-inflammatory cytokines^47,48^.

Gliosis, as shown by increased GFAP staining intensity, was increased at all anatomical locations in all TBI groups compared to the sham (non-injured) rats as expected in response to this type of traumatic injury for tissue repair. Both of the treatment groups showed a small increase in GFAP intensity at 6 weeks compared to the TBI only group (p < 0.05) but only in the cortex. However, the microglial at 6 weeks response was significantly increased in the cortex, hippocampus and thalamus of the TBI-LC group compared to the TBI only (p < 0.05), corresponding to the time that there were considerable improvements in short memory function during novel objective recognition test. Microglia are thought to have an important role as immune modulators for CNS tissue repair^47–49^ and the elevated levels of activated microglia may have a beneficial effect on the tissue at these later time points through the release of growth factors or anti-inflammatory cytokines.

After acute injury, there is an increase in energy demand. As such, most mitochondrial respiratory chain functional markers OXPHOS complexes were significantly increased within 24 h as shown here in the TBI brains. However, such response also produces more by-product reactive oxygen species increasing oxidative stress as shown in this study. The endogenous antioxidants would counteract such increases in ROS; however during TBI, antioxidants such as MnSOD can become inactivated or overwhelmed with the occurrence of ROS overproduction^50^. Additionally, trauma can disrupt the fine compartments of a cell resulting in selective antioxidants adopting a pro-oxidative role serving to promote the synthesis of oxidative free radicals^51^. In this study, although MnSOD protein level was not significantly changed in the TBI rats, oxidative stress markers were increased at 24 h. This imposes more pressure on already vulnerable mitochondria, which can be directly damaged by oxidative stress. Indeed, oxidative stress plays a major role in TBI-induced tissue damage and energy failure due to its direct effect on the ATP synthesis site in the mitochondria^52,53^. As such, at 6 weeks, there was some reduction in OXPHOS complexes I and V levels although not statistically significant. This was associated with increased ROS levels suggesting mitochondrial damage. It is worth noting that western blotting can quantify the protein level of each complex which does not reflect mitochondrial activity. The by-product ROS is only suggestive of mitochondrial function.

The damaged mitochondria would activate the mitophagy process to repair (fusion) and eliminate (fission) damaged mitochondrial fragment which is protective to neural injury^54,55^. Neither mitophagy fusion and fission markers in this study were significantly changed by brain injury. This may be due to the acute time point. Nevertheless, oxidative stress can cause the cell membranes to break down inactivating critical membrane pumps, altering gene regulation, and impairing signal transduction, consequently hindering the normal function of the cells and rendering them susceptible to cell death^56^. It is reflected by TUNEL staining that there was an increase in DNA damage in cortex, hippocampus and thalamus. Increases in DNA damage might not lead to cell death immediately, this is reflected by unchanged caspase-3 level at 24 h^57^. From our result, it shown that apoptosis did not occur at 24 h in TBI group, possibly protected by stable mitochondrial antioxidant MnSOD^58^. As a result, although there was only a small contusion at the cortex epicentre at 24 h, a larger size cavity was found at 6 weeks, suggesting ongoing cell death after the initial injury but time of apoptosis is unknown. A study from Clark *et al*. showed that apoptosis initiated one to three days after TBI^59^. In that study, TBI insulted a greater injury as indicated by 2.5 mm depth of injury causing significant damage of cerebral cortex ipsilateral to the injury^59^. Additionally, significant motor and sensory deficits, as well as some level of cognitive deficit (although not statistically significant), were observed at 24 h post injury.

Clinically, mild TBI results in a broad spectrum of injuries from minimal neurometabolic changes with rapid recovery to irreversible structural damage resulting in persistent functional deficits^60^. The current management strategies in place exclusively cater to one end of this range neglecting the more severe cases of mild TBI. Mitochondrial dysfunction is a common factor amongst numerous biochemical cascades initiated by TBI; linking the primary cellular changes to the eventual neuronal damage and possible cell death^61,62^. This is particularly true for neuronal cells of the brain given their high energy demands^63^. With such an extensive and crucial action in the brain, it is not surprising mitochondrial dysfunction has been identified as a significant influence in the cell death of numerous neurological disorders^64–67^. Therefore, in this study, we have tested two antioxidants that have been shown to be protective in different organs against oxidative-stress induced damage.

L-Carnitine assists in the transportation of long-chain fatty acids into the mitochondrion for energy synthesis^68–70^. L-Carnitine has a number of therapeutic applications, such as that in type 2 diabetes^71,72^, myocardial infarction^73,74^, and kidney disease^75^. When TBI happens, the limited glucose storage is depleted rapidly and the brain is forced to utilise amino acids as an energy substrate as ATP is essential for the maintenance of general brain function^76^. L-Carnitine has been found to improve mitochondrial function in conditions of oxidative stress by detoxifying free-radicals and enhancing the activity of endogenous antioxidants^77^. In this study, L-Carnitine reduced oxidative stress and increased endogenous antioxidant MnSOD, with enhanced mitochondrial functional makers at 24 h post injury. This may directly contribute to the neuroprotective effect, where ameliorated tissue injury and neurological functional deficit were found in TBI rats treated with L-Carnitine immediately after the injury. Such protective effect might be delayed later than 24 h post injury.

Exendin-4 is an agonist of the glucagon-like peptide-1 receptor, which is currently prescribed for the treatment of type 2 diabetes and obesity^78,79^. However, several studies have also found its neuroprotective abilities in various models of neurological disorders^21,22,80^ including a mouse model of blast-TBI^81,82^. In the current study, protective effects were shown in Exendin-4 treated rats with TBI at both 24 h and 6 weeks with similar effects to L-Carnitine on the cortex tissue and ameliorated neurological dysfunction and oxidative stress markers after the traumatic force. Whilst the beneficial impact of Exendin-4 on mitochondria was suggested in a study on retinal ganglion cells injury, Exendin-4 only induced a non-significant increase in TOM20 and fusion marker Opal-1 at 6 weeks^80^. DNA damage was reduced significantly in the cerebral cortex compared to TBI group at 24 h suggesting that Exendin-4 is protective through preventing initiation of apoptosis within 24 h post injury. However, we need to acknowledge that pinpointing the components of mitochondrial function that are impaired is extremely difficult. The negative finding in mitochondrial markers does not exclude its positive impact on mitochondrial integrity and function to support rapid repair after the injury.

In summary, the outcome of both treatment options in this study, L-Carnitine and Exendin-4, supports their strong potential to be repurposed in reducing brain tissue damage and functional decline due to mild TBI in humans. A future long-term study for a minimum of 6 months is required to investigate whether such treatments can prevent long-term posttraumatic neurodegeneration.

## Electronic supplementary material

Supplementary information
