# Supplementary material for: L-Carnitine and extendin-4 improve outcomes following moderate brain contusion injury
Source: Sci Rep. 2018 Jul 25;8:11201. doi: 10.1038/s41598-018-29430-6 (PMC6060156; doi:10.1038/s41598-018-29430-6)
Supplement: Supplementary file 1 — Supplementary information [file 41598_2018_29430_MOESM1_ESM.pdf]

L-Carnitine and extendin-4 improve outcomes following moderate brain contusion injury.

Hui Chen [1†], Yik Lung Chan [1†], Claire Linnane [1], Yilin Mao[1], Ayad G Anwer[2], Arjun Sapkota [1], Tiara F Annissa[3], George Herok [1], Bryce Vissel[4,5], Brian G Oliver [1], Sonia Saad[6], Catherine A Gorrie *[1]

Supplementary information

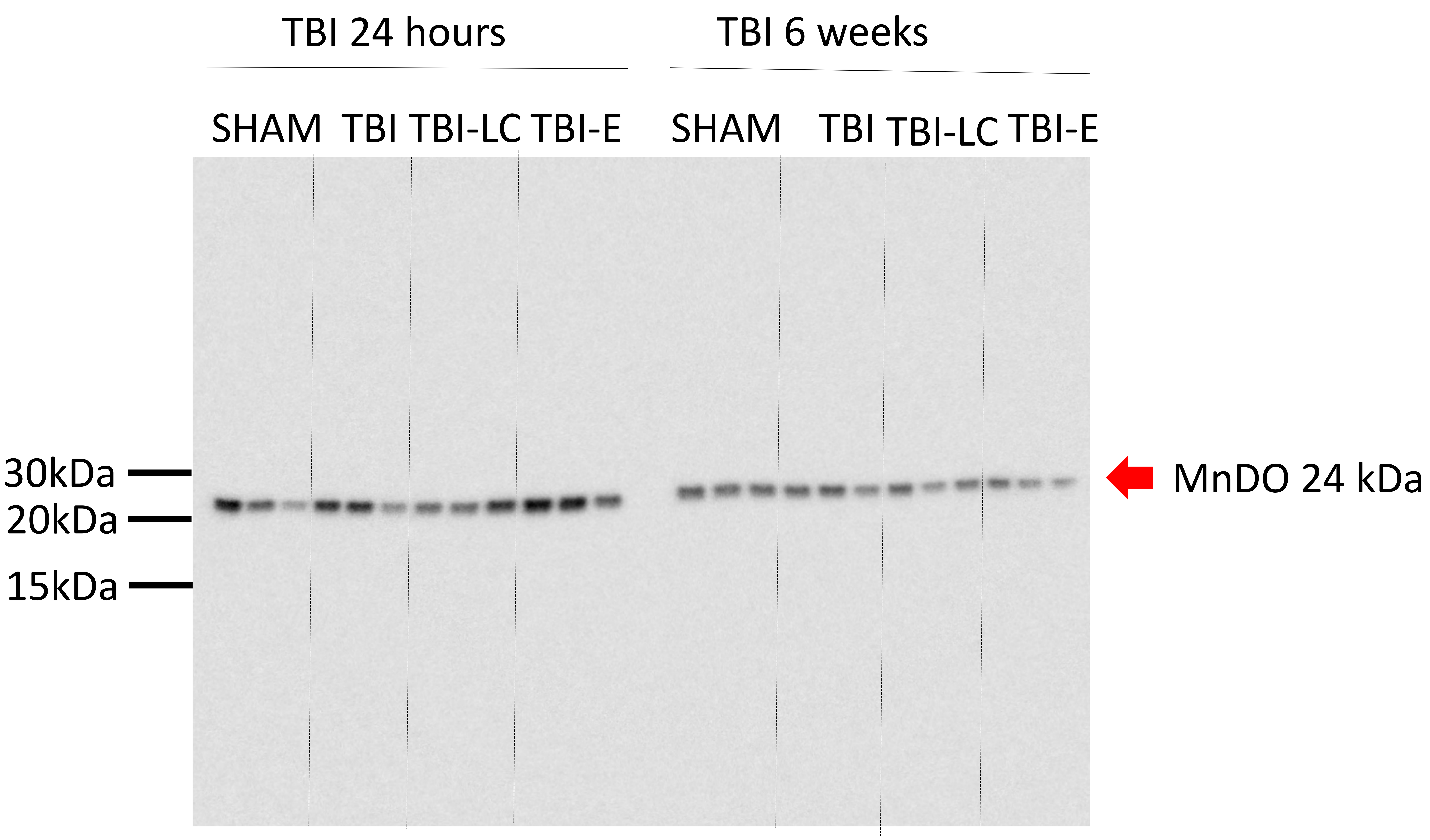

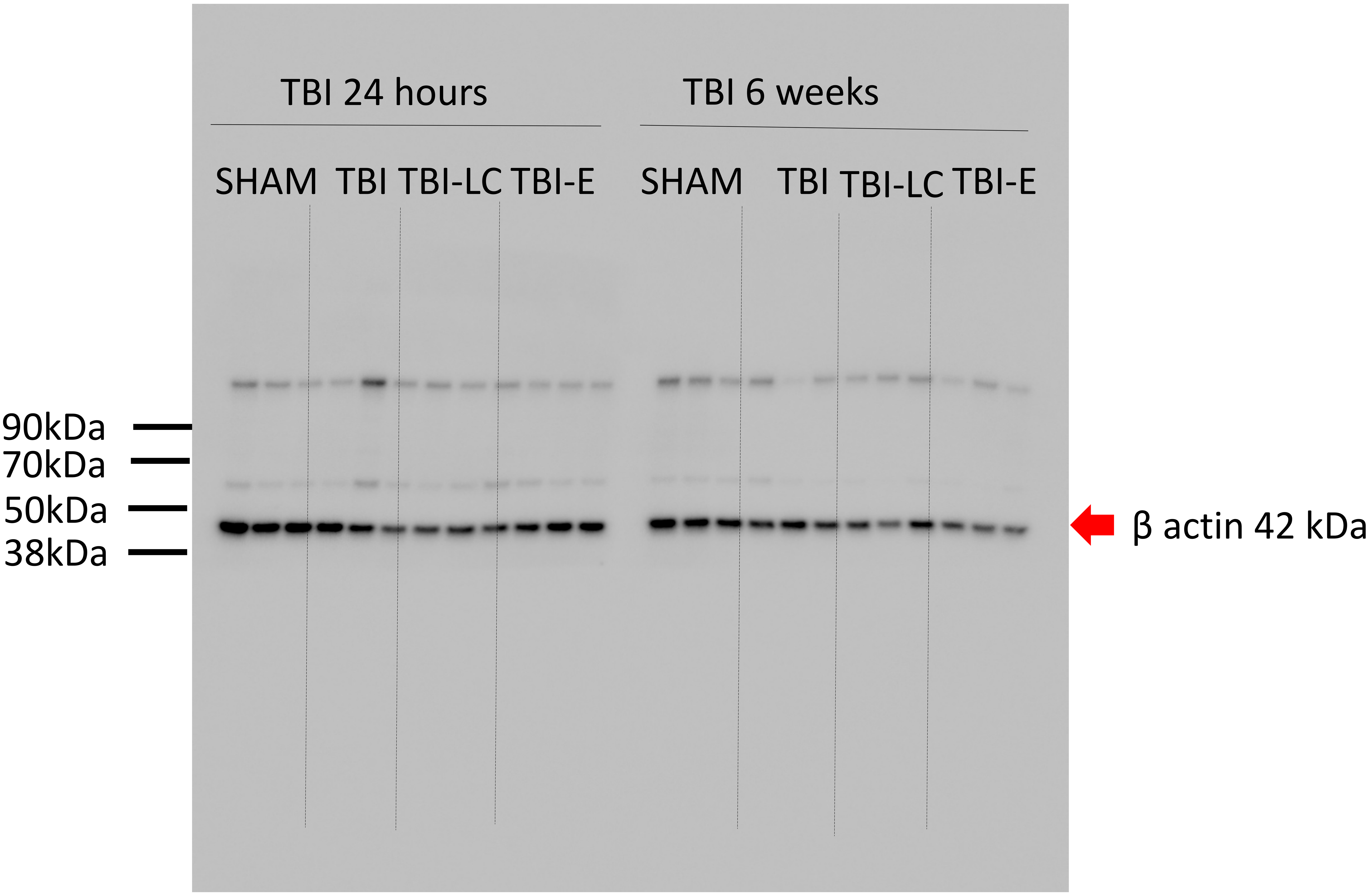

Supplementary figure 1. Original blot of Figure 5C and 5d.

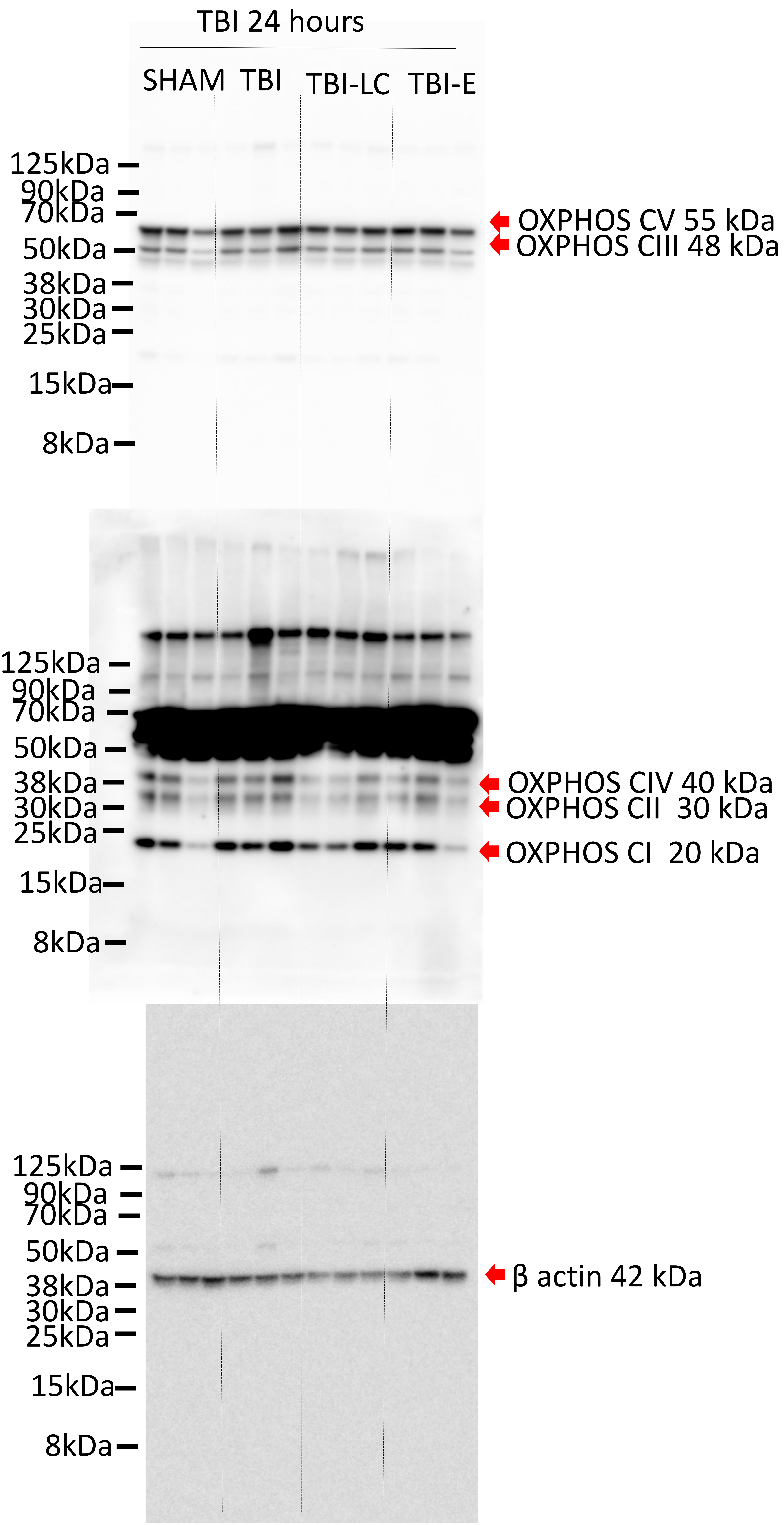

Supplementary figure 2. Original blot of Figure 6a, OXPHOS protein at 24 hours

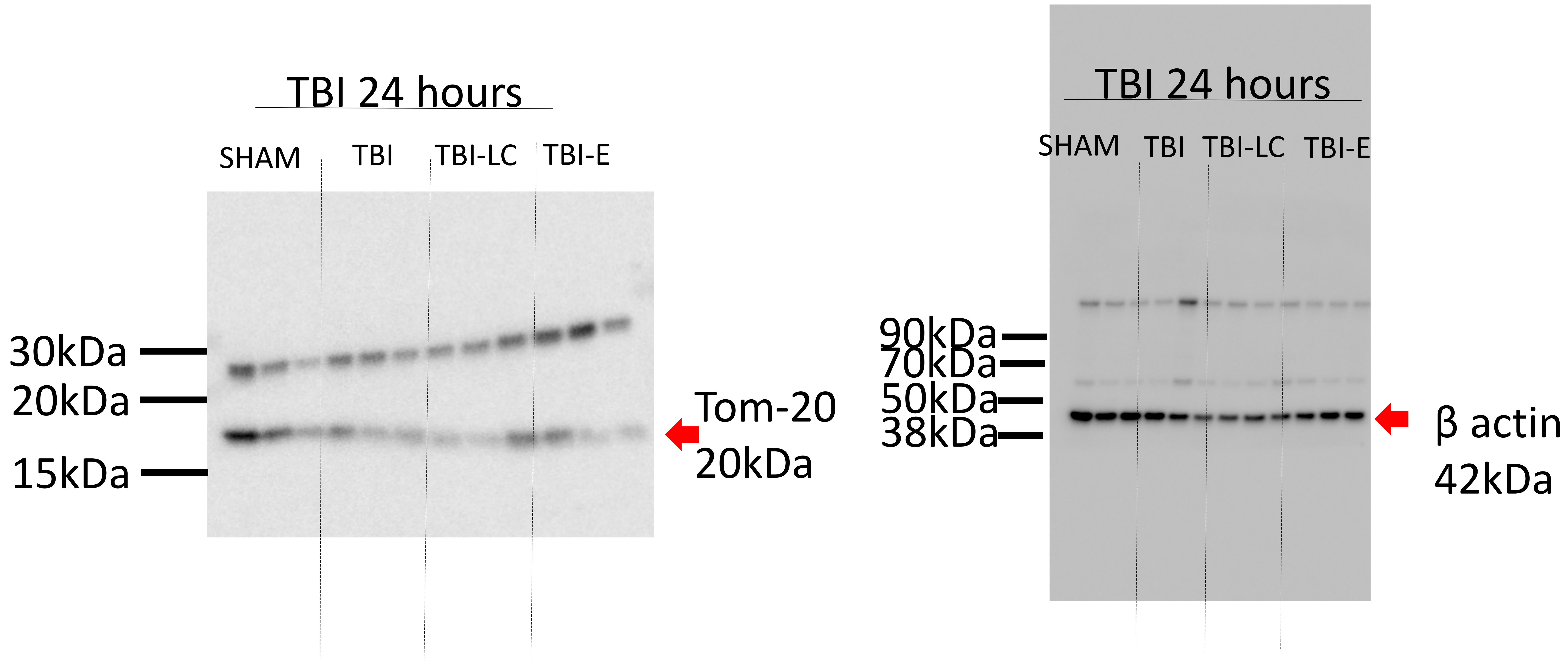

Supplementary figure 3. Original blot of Figure 6b

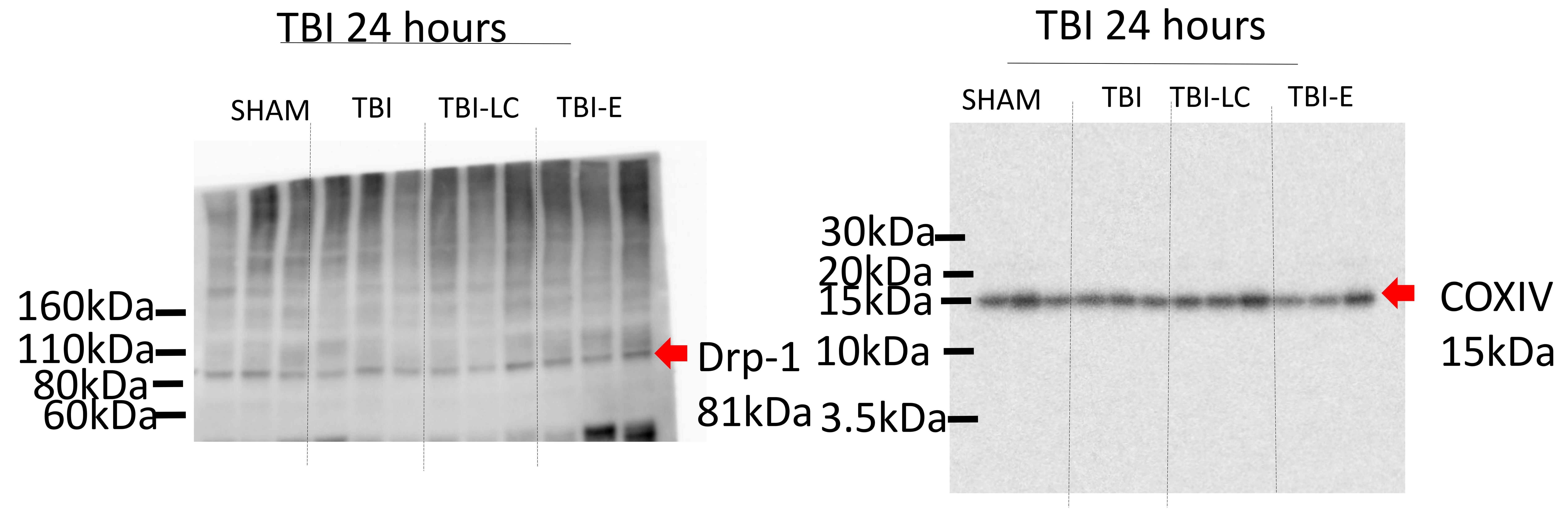

Supplementary figure 4. Original blot of Figure 6c

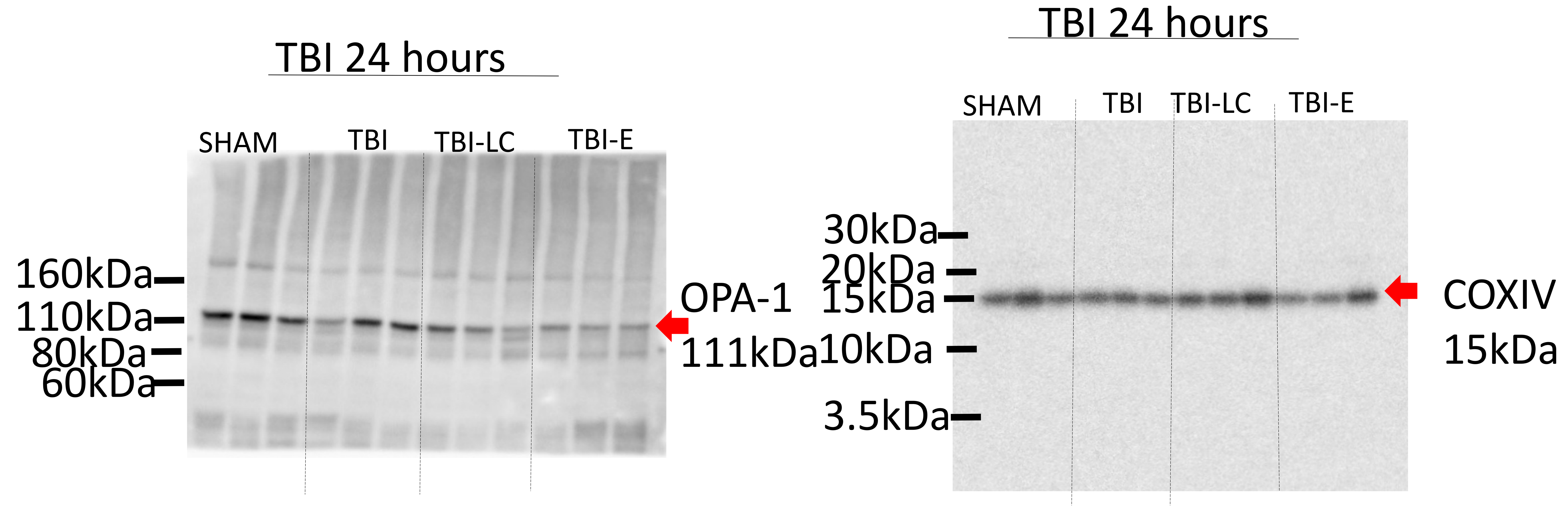

Supplementary figure 5. Original blot of Figure 6d

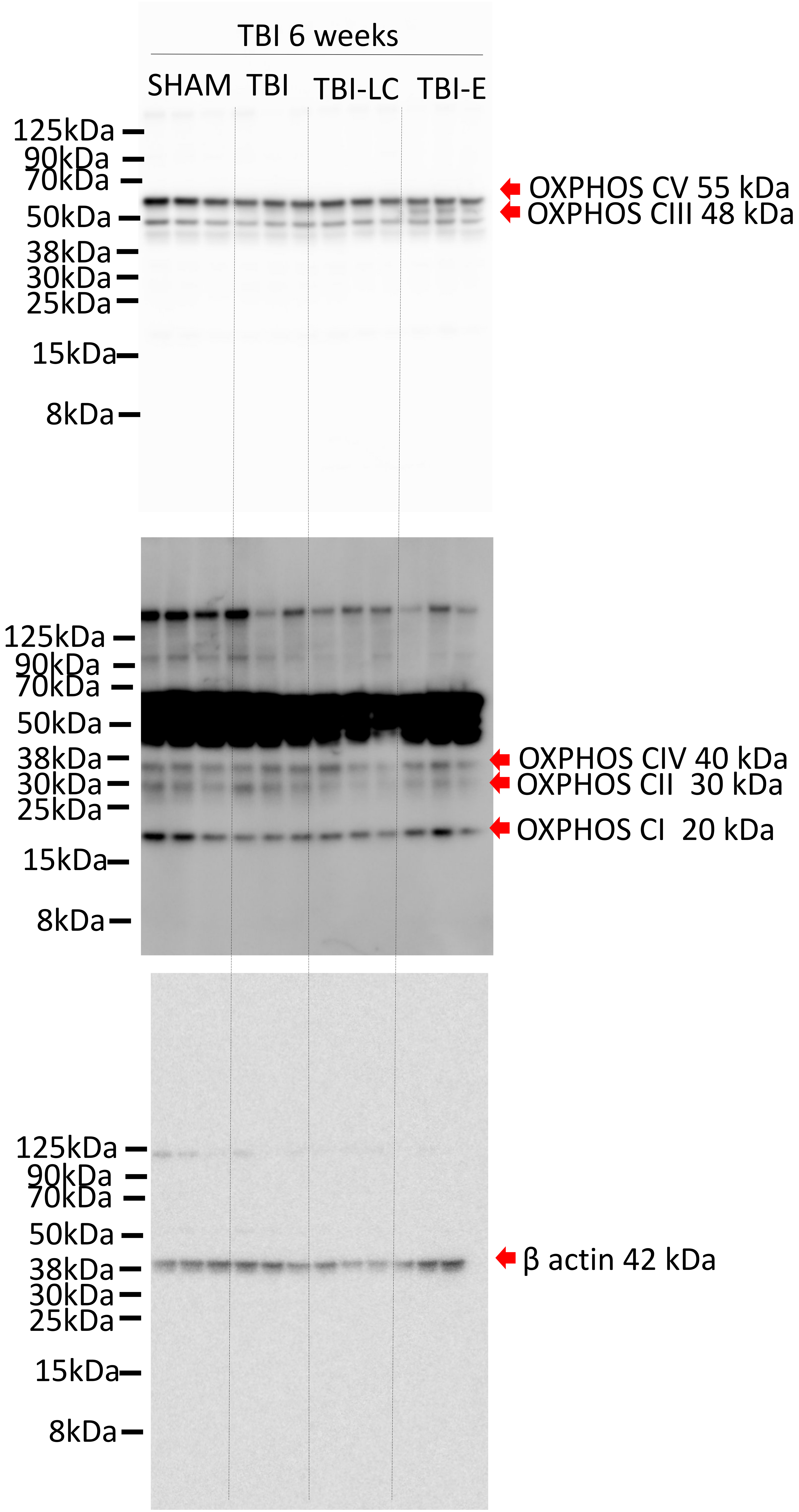

Supplementary figure 6. Original blot of Figure 6e

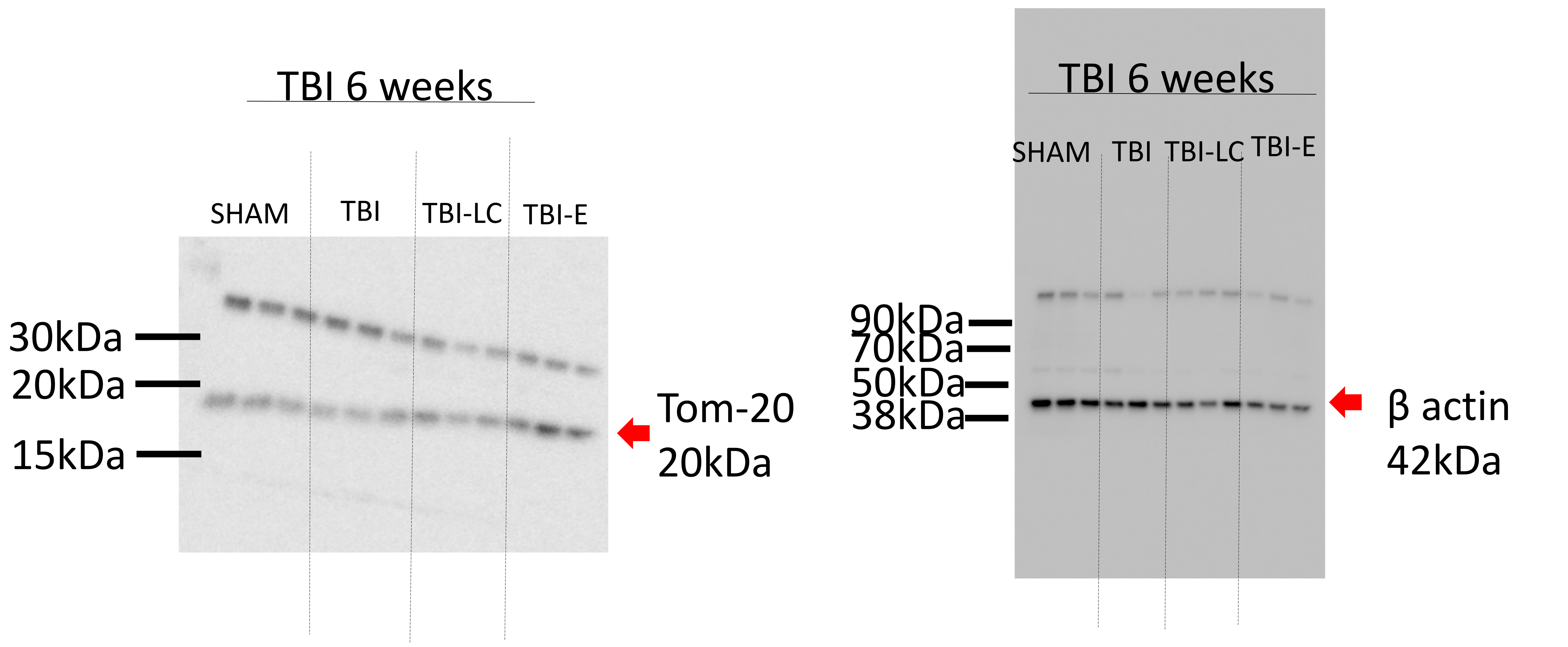

Supplementary figure 7. Original blot of Figure 6f

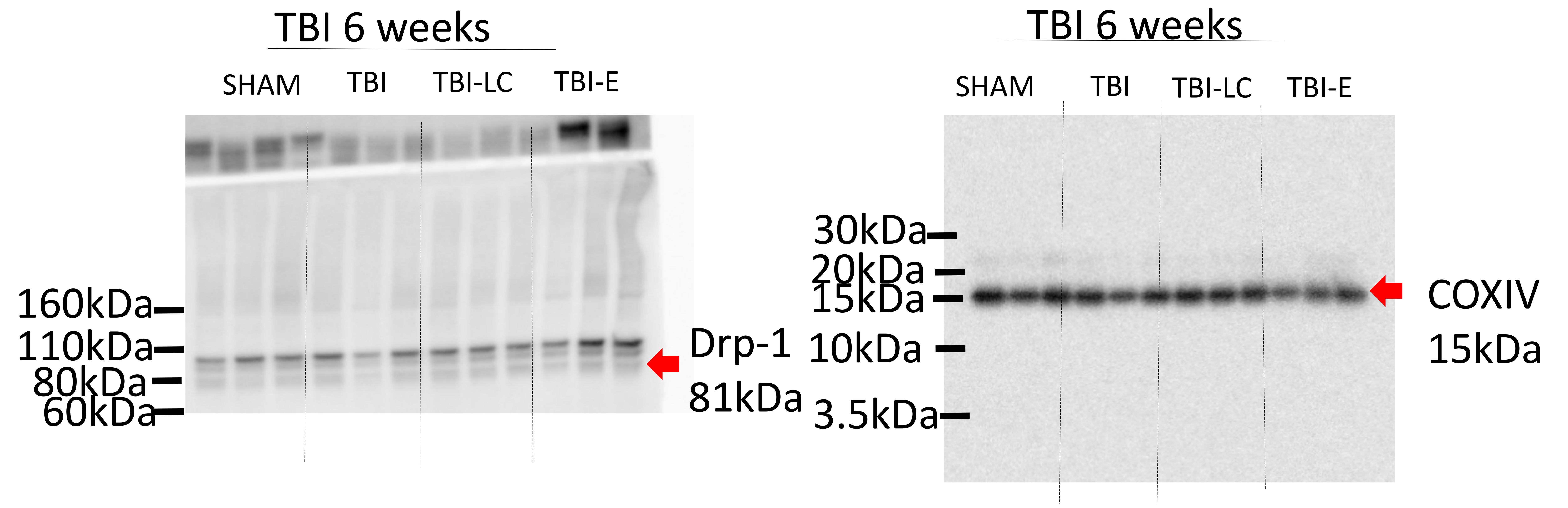

Supplementary figure 8. Original blot of Figure 6g

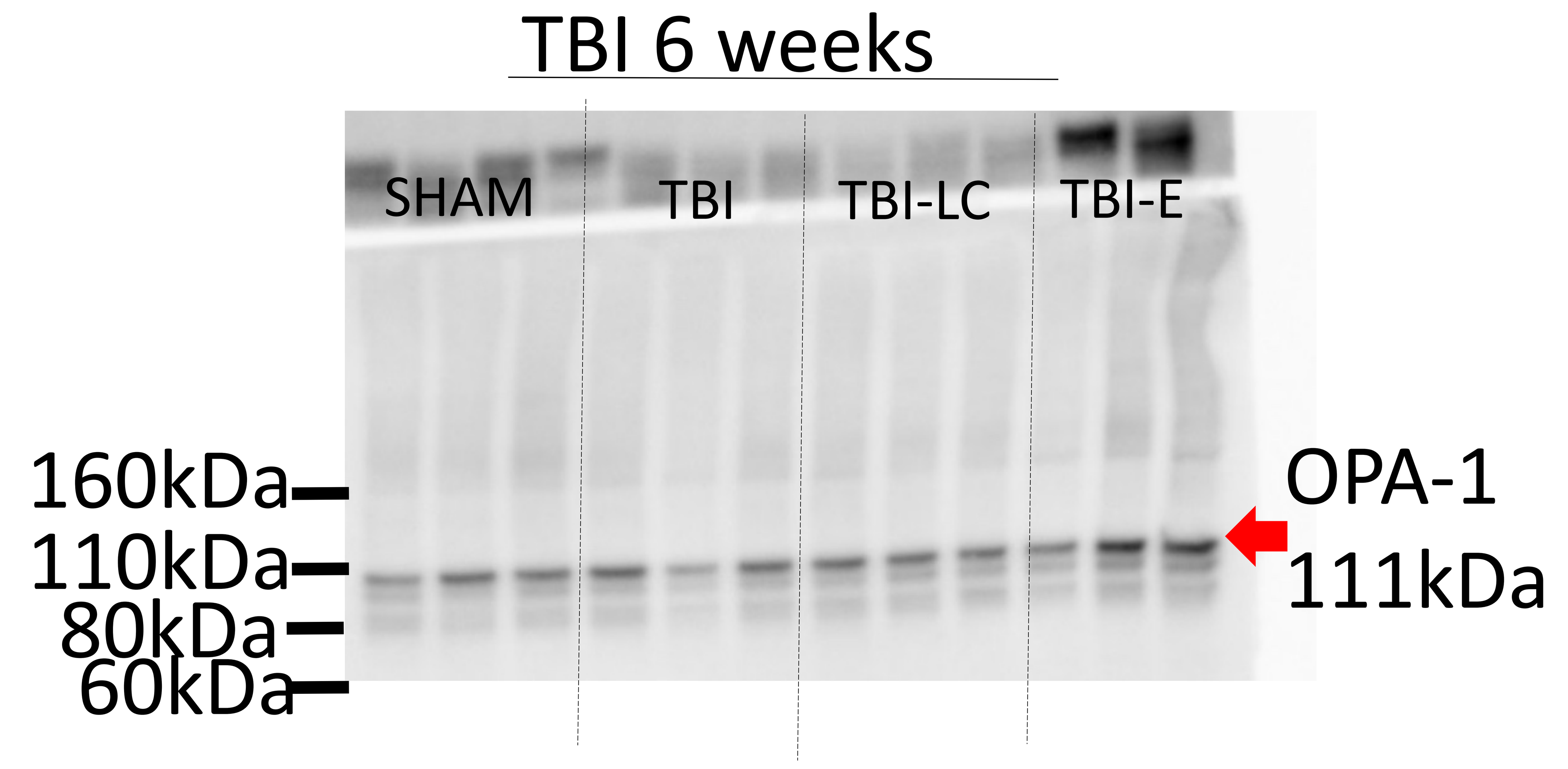

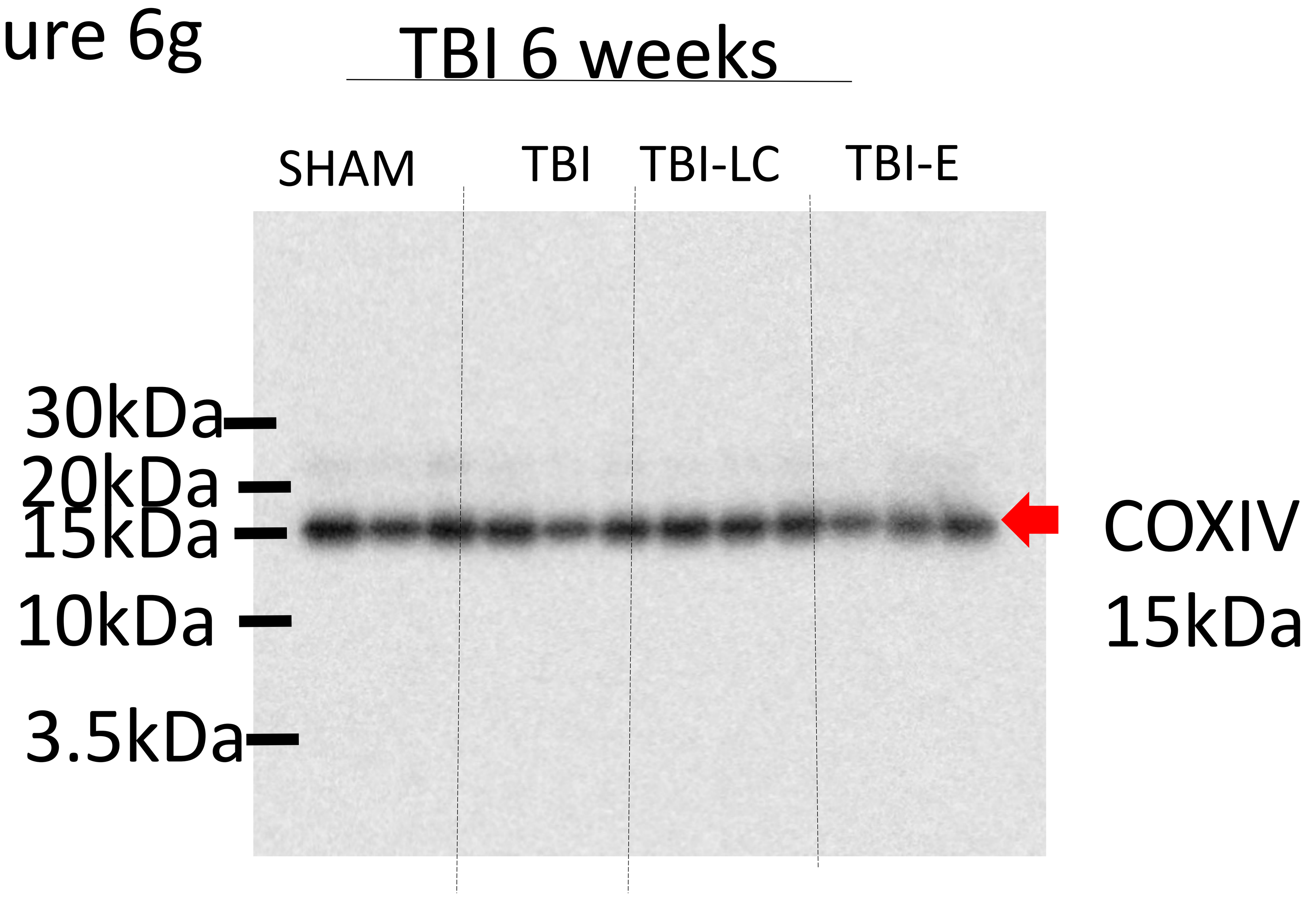

Supplementary figure 9. Original blot of Figure 6h
